# Tumor Exosomal L1 Cell Adhesion Molecule Promotes Brain Metastasis of Lung Cancer

**DOI:** 10.34133/research.1126

**Published:** 2026-02-10

**Authors:** Dong Ha Kim, Chae Won Lee, Yun Jung Choi, Da-Som Kim, Kyosun Ban, Juhyeon Hong, Gyeong Joon Moon, Sang-Yeob Kim, Chan-Gi Pack, In-Jeoung Baek, Jin-Yong Jeong, Dong-Cheol Woo, Ji-Hye Oh, Chang Ohk Sung, Kyunggon Kim, Hyun-Yi Kim, Hae-Yun Jung, Wonjun Ji, Min Jee Kim, Chang Min Choi, Jae Cheol Lee, Jin Kyung Rho

**Affiliations:** ^1^ Asan Institute for Life Sciences, Asan Medical Center, University of Ulsan College of Medicine, Seoul, South Korea.; ^2^ Department of Biochemistry and Molecular Biology, Brain Korea 21 Project, Asan Medical Center, University of Ulsan College of Medicine, Seoul, South Korea.; ^3^ Center for Cell Therapy, Asan Medical Center, University of Ulsan College of Medicine, Seoul, South Korea.; ^4^ Department of Convergence Medicine, Asan Medical Center, University of Ulsan College of Medicine, Seoul, South Korea.; ^5^ Department of Cell and Genetic Engineering, Asan Medical Center, University of Ulsan College of Medicine, Seoul, South Korea.; ^6^ MR/CT/US Core Laboratory, Asan Medical Institute of Convergence and Technology, Asan Medical Center, University of Ulsan College of Medicine, Seoul, South Korea.; ^7^ Bioinformatics Core Laboratory, Convergence Medicine Research Center, Asan Medical Center, University of Ulsan College of Medicine, Seoul, South Korea.; ^8^ Department of Digital Medicine, Brain Korea 21 Project, Asan Medical Center, University of Ulsan College of Medicine, Seoul, South Korea.; ^9^ NGeneS Inc., Asna-Si, Gyeonggi-do, South Korea.; ^10^ Division of Radiation Biomedical Research, Korea Institute of Radiological and Medical Sciences, Seoul, South Korea.; ^11^ Department of Pulmonary and Critical Care Medicine, Asan Medical Center, University of Ulsan College of Medicine, Seoul, South Korea.; ^12^ Department of Oncology, Asan Medical Center, University of Ulsan College of Medicine, Seoul, South Korea.

## Abstract

Brain metastasis (BrM) is a common occurrence in lung cancer and substantially worsens the prognosis due to the blood–brain barrier (BBB), which restricts drug entry into the brain. Here, we found that exosomes secreted by lung cancer cells that had acquired epidermal growth factor receptor tyrosine kinase inhibitor resistance and undergone epithelial–mesenchymal transition (osimertinib- and WZ4002-resistant H1975) exhibited enhanced brain-specific distribution and a concomitant increase in BrM compared with exosomes from parental H1975 cells. To identify exosomal mediators of this phenotype, liquid chromatography-tandem mass spectrometry-based proteomic analysis was performed. Exosomes derived from resistant cell lines exhibited distinct protein profiles relative to parental cells, with 744 exosomal proteins significantly altered (fold change ≥ 2; *P* ≤ 0.05). Prioritization of membrane proteins and ligand–receptor interaction analysis identified ITGAV (integrin αV), ITGB3 (intergrin β3), and L1CAM (L1 cell adhesion molecule) as candidates interacting with brain-specific ligands, including neural cell adhesion molecule 1 (NCAM1) and contactin 2. Validation of exosomal association by Western blotting identified ITGB3 and L1CAM as final candidates. Subsequent functional modulation studies demonstrated that exosomal L1CAM plays a dominant role in brain distribution and metastatic progression. Exosomal L1CAM increased BBB permeability by disrupting endothelial tight-junction integrity both in vitro and in vivo. This effect was associated with the involvement of NCAM1 on BBB endothelial cells, as suggested by an exosomal L1CAM masking experiment. Clinically, exosomal L1CAM demonstrated diagnostic potential for BrM (area under the curve [AUC] = 0.80), and a combined exosomal L1CAM/ITGB3 panel significantly improved diagnostic accuracy (AUC = 0.98). Collectively, these findings identify exosomal L1CAM as a key regulator of brain-specific metastasis and support the clinical utility of the L1CAM/ITGB3 panel as a noninvasive biomarker for BrM in lung cancer.

## Introduction

Lung cancer is one of the malignancies with the highest propensity to metastasize to the brain, accounting for approximately 40% to 50% of all cases of brain metastasis (BrM) [1]. Conversely, an estimated 10% to 20% of patients with non-small-cell lung cancer (NSCLC) present with BrM at the time of initial diagnosis [2,3]. The development of BrM in lung cancer is associated with a significantly worsened prognosis, often resulting in substantially reduced survival despite modern therapies [4]. Therefore, early diagnosis of BrM is of utmost importance. Detecting metastatic spread while lesions are still small and asymptomatic can facilitate timely intervention and improve patient outcomes. Despite its clinical significance, the mechanisms underlying brain-specific metastasis in lung cancer remain poorly understood. A significant challenge in this context is the restrictive nature of the blood–brain barrier (BBB), which tightly regulates the entry of cells and macromolecules into the central nervous system [5]. Consequently, metastatic dissemination to the brain requires cancer cells to overcome this highly selective vascular barrier.

Cancer metastasis to distant organs is not a random process but rather an organ-selective phenomenon. In 2005, Kaplan et al. [6] first demonstrated that primary tumors can recruit bone-marrow-derived cells to distant tissues, thereby creating a permissive niche that promotes subsequent tumor cell engraftment. One of the most important mediators of this process is tumor-derived exosomes. Exosomes are nanosized extracellular vesicles that contain proteins, lipids, and nucleic acids, which are released into the bloodstream and play a critical role in cell-to-cell communication [7–9]. In particular, tumor-derived exosomes can modulate the immune response, angiogenesis, and stromal characteristics in target organs, thereby priming a prometastatic microenvironment [10–12]. A landmark study by Hoshino et al. [13] provided direct evidence that tumor exosomes dictate the organotropism of metastasis. Specifically, the author demonstrated that exosomes derived from distinct tumor types carry unique combinations of integrin proteins on their surface, which determine their preferred target organs. Importantly, these exosomes exhibit site specificity that is dependent on their integrin profile, with integrin β3 (ITGB3) being specific to the brain [13–15]. Exosomal ITGB3 has garnered attention as a potential biomarker for predicting BrM. However, its sensitivity and specificity as a standalone marker are limited, particularly in heterogeneous patient populations. Therefore, it is important to integrate it into a multimarker panel and conduct additional clinical validation to enhance its applicability in predicting BrM [15].

L1 cell adhesion molecule (L1CAM) is a protein belonging to the immunoglobulin superfamily that is aberrantly expressed in numerous aggressive cancers. It is now recognized as a critical factor in promoting the early growth of BrM [16,17]. L1CAM is a 200- to 220-kDa transmembrane glycoprotein, consisting of an extracellular region that contains 6 immunoglobulin-like domains and 5 fibronectin type III repeats, a single transmembrane segment, and a short cytoplasmic tail [16,18]. This modular structure enables L1CAM to mediate both homophilic binding between cells (L1CAM–L1CAM) and heterophilic binding with various ligands (e.g., integrins, neural cell adhesion molecule [NCAM] family, neurocan, neuropilin-1, and CD24) [18–20]. Through these interactions, L1CAM activates signaling pathways such as nuclear factor κB and yes-associated protein, which promote tumor cell migration, survival, and growth in BrM [21,22]. L1CAM is essential for colonization of the cerebral microenvironment, facilitating the attachment of circulating tumor cells to brain capillaries [23], mimicking pericytes [21], and initiating metastatic outgrowth [24]. Clinically, L1CAM expression is associated with a more aggressive tumor phenotype and correlates with metastatic relapse, making it an attractive target for therapeutic intervention [25]. Ongoing studies are exploring how L1CAM interacts with organ-specific factors to promote metastasis and how disrupting these interactions may prevent or treat brain metastatic disease [20].

In this study, we demonstrate that exosomal L1CAM, which is abundant in epithelial–mesenchymal transition (EMT)-resistant lung cancer cells, serves as a key mediator of BBB dysfunction and brain-specific metastasis. Through analyses of BBB permeability, proteomic and transcriptomic profiling, and clinical validation, we demonstrated that exosomal L1CAM promotes BrM by enhancing endothelial cell permeability and creating a favorable environment for metastatic spread. In addition, we propose a biomarker panel consisting of exosomal ITGB3 and L1CAM, which exhibits high diagnostic accuracy for the detection of BrM in patients with lung cancer. Our findings provide mechanistic and clinical insights into exosome-mediated brain tropism and suggest novel therapeutic and diagnostic approaches for metastatic lung cancer.

## Results

### Optimization and characterization of exosome isolation methods

In a previous study, we established third-generation epidermal growth factor receptor tyrosine kinase inhibitor (EGFR-TKI)–resistant cell lines (osimertinib- and WZ4002-resistant H1975 [H1975/OR and H1975/WR, respectively]) from the NSCLC cell line H1975. We designated these cells as EMT-associated resistant cells based on their phenotypic changes, including a transition to a spindle-shaped morphology, reduced epithelial marker proteins, induction of vimentin expression, and enhanced cell motility [26]. We compared ultracentrifugation and tangential flow filtration (TFF) for exosome isolation from conditioned media and ultimately used TFF for the functional validation of exosomes. The isolated exosomes were thoroughly characterized for size distribution, quantity, morphology, and marker protein expression (Fig. S1A to D). NanoSight analysis indicated that the total number of exosome particles secreted by EMT-associated resistant cells (H1975/OR and H1975/WR) was lower than that secreted by parental H1975 exosomes. Despite the reduced number of secreted exosomes, the protein content per particle was significantly higher in exosomes derived from the EMT-associated resistant cells (Fig. S1E to H).

### Exosomes from EMT-associated resistant cells induce BrM

**Fig. 1.**
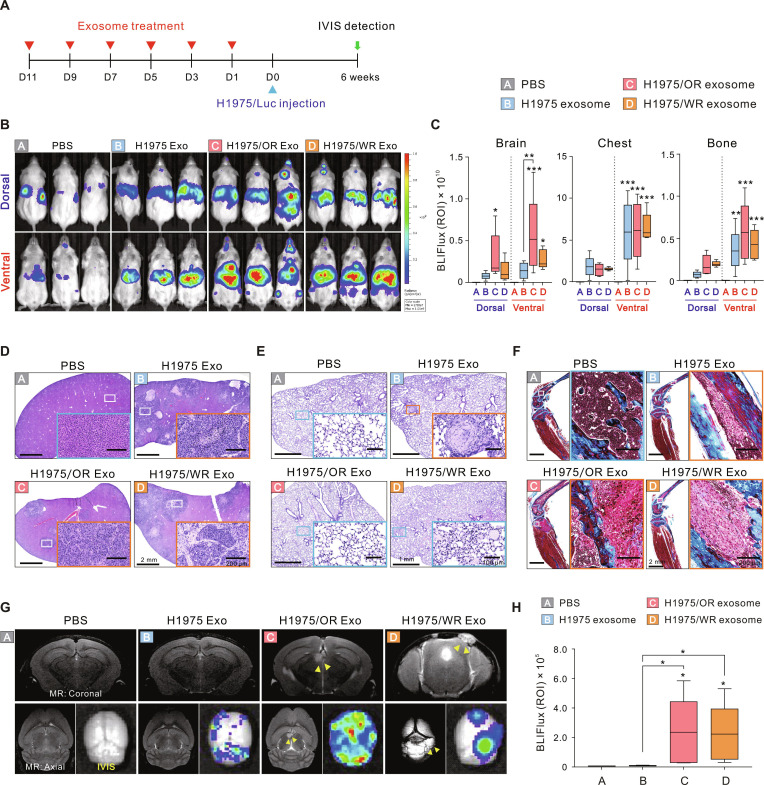
Exosome education model for confirming metastasis patterns to specific organs. (A) Schematic diagram of the lung-cancer-cell-derived exosome education model for metastasis detection. D, day. (B) Representative IVIS images of mice injected with H1975/Luc cells following each exosome education. (C) IVIS values quantified in the dorsal and ventral regions, separated into brain, chest, and bone compartments. BLI flux refers to bioluminescence imaging flux, defined as the total photon flux (in photons per second) measured within a specified region of interest (ROI) using IVIS imaging. Exo, exosome. (D to F) Representative images of hematoxylin and eosin staining of mouse liver (D) and lung (E) and Masson’s trichrome staining of bone (F). Blue squares represent nonmetastatic sites, and orange squares represent metastatic sites. (G) Representative images of mouse brain MRI and IVIS. Yellow arrows indicate tumor locations. (H) Quantified BrM levels based on IVIS images. Statistical significance for metastasis levels is indicated by an asterisk. Data are presented as the means ± SD (*n* = 5 mice per group). *P < 0.05, **P < 0.005, and ***P < 0.0005.

To investigate whether exosomes derived from EMT-associated resistant cells (H1975/OR and H1975/WR) affect organotropic metastasis, we performed exosome education experiments. Nonobese diabetic severe combined immunodeficient gamma (NSG) mice received intracardiac injection of each exosome every 2 days for a total of 6 injections, followed by intracardiac injection of luciferase-labeled H1975 (H1975/Luc) cells to facilitate systemic circulation of lung cancer cells. Metastatic progression was monitored using the In Vivo Imaging System (IVIS) (Fig. 1A). IVIS images were captured from both dorsal and ventral regions, and the extent of metastasis was quantified separately in the brain, chest, and bone (hind limb) compartments (Fig. 1B). Notably, exosomes derived from EMT-associated resistant cells (H1975/OR and H1975/WR) significantly enhanced BrM compared to the parental H1975 exosomes, with the most pronounced effect observed in the H1975/OR exosome-treated group (Fig. 1C). BrM was detected early (days 5 to 10) following the injection of H1975/Luc cells, particularly in the H1975/OR and H1975/WR exosome-treated groups, and progressively worsened thereafter (Fig. S2). The early detection of brain metastatic lesions supports the hypothesis that exosome-mediated formation of a metastatic niche may play a crucial role in the establishment of early BrM.

Histopathological examination further corroborated these findings, revealing tumor lesions in the liver and bone of all exosome-treated groups, whereas no lesions were detected in the phosphate-buffered saline (PBS)-treated control (CON) group. In the lungs, tumor lesions were observed exclusively in the group treated with H1975 exosomes (Fig. 1D to F). Notably, tumor lesions were identified on brain magnetic resonance imaging (MRI) only in the EMT-associated resistant cell-derived exosome groups (H1975/OR and H1975/WR), and IVIS values were significantly elevated in the extracted brains (Fig. 1G and H). Collectively, these data suggest that EMT-associated resistant cell-derived exosomes may enhance the metastatic capacity of tumor cells by establishing a brain-specific metastatic niche.

### Exosomes derived from EMT-associated resistant cells demonstrate increased uptake into the brain

**Fig. 2.**
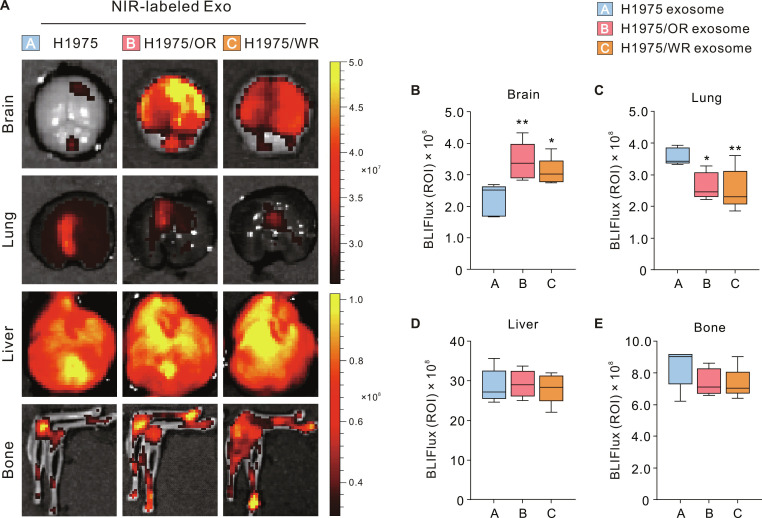
Brain-specific biodistribution of EMT-associated resistant cell-derived exosomes. Biodistribution of exosomes derived from human lung cancer cell lines. (A) Representative IVIS images of the brain, lung, liver, and bone following the intracardiac injection of individual NIR-labeled exosomes in mice. (B to E) Quantification of exosome biodistribution levels by IVIS in each organ. Statistical significance for biodistribution levels is indicated by an asterisk. Data are presented as the means ± SD (*n* = 5 mice per group). *P < 0.05 and **P < 0.005.

To determine whether BrM-promoting exosomes preferentially accumulate in future metastatic sites, we intracardially injected near-infrared (NIR)-labeled H1975, H1975/OR, and H1975/WR exosomes into mice. At 24 h postinjection, we extracted samples from the brain, lung, liver, and hind limb bone, which are known high metastatic sites for lung cancer. We utilized IVIS imaging to assess the biodistribution and uptake of exosomes in each distant organ (Fig. 2A). Quantifying the biodistribution of exosomes in the brain, we found that exosomes from H1975/OR and H1975/WR cells were significantly higher compared to the H1975 exosomes, with the increase being more pronounced for H1975/OR exosomes (Fig. 2B). In contrast, the biodistribution of H1975/OR and H1975/WR exosomes in the lungs was significantly reduced compared to H1975 exosomes (Fig. 2C). No significant differences were observed between the groups for the liver and bone (Fig. 2D and E). These results suggest that exosomes derived from EMT-related resistant cells may promote metastasis due to their specific biodistribution to the brain.

### Exosomal membrane proteins ITGB3 and L1CAM interact with cerebral cortex proteins

**Fig. 3.**
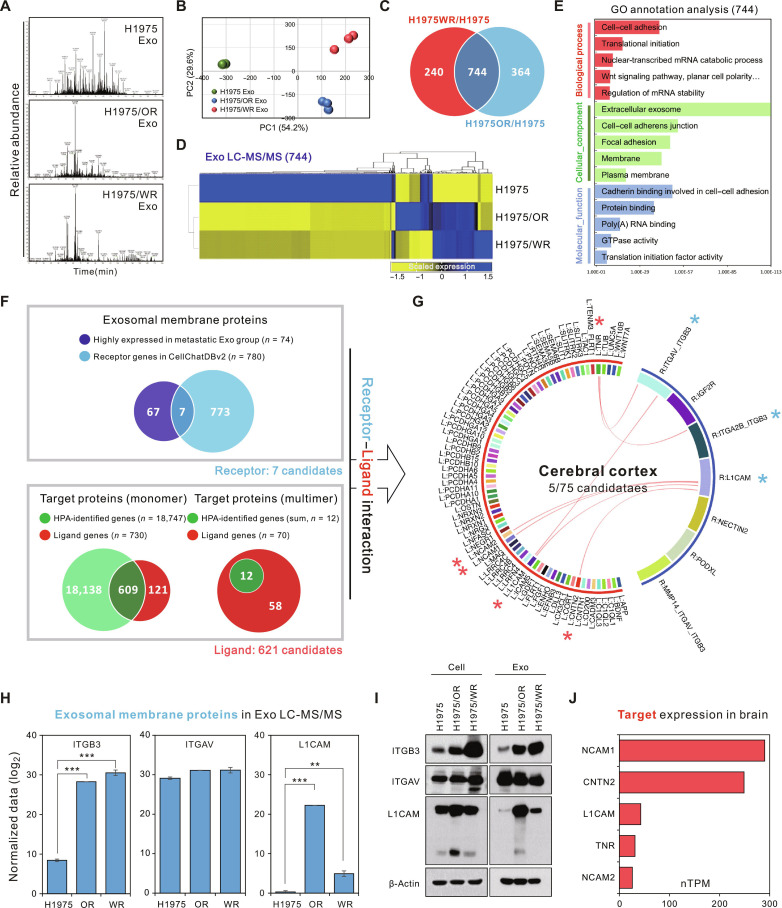
Identification of BrM-associated exosomal L1CAM and ITGB3 via proteomic analysis. LC-MS/MS proteomics analysis was conducted using exosomes collected from nonmetastatic H1975 as well as metastatic H1975/OR and H1975/WR cell lines. (A) Fragmentation patterns of H1975, H1975/OR, and H1975/WR exosomal peptides by LC-MS/MS. (B) PCA plot of the exosome LC-MS/MS. (C) Venn diagram shows the number of commonly expressed exosomal proteins between the groups. (D) Heatmap analysis shows the expression levels of 744 common exosomal proteins that were significantly up- and down-regulated more than 2-fold (*P* < 0.05) between the groups. (E) GO enrichment analysis was performed on the basis of the 744 genes that exhibited significant increases and decreases in each group. GTPase, guanosine triphosphatase. (F and G) Inference model for the interaction network between exosomal membrane proteins and target proteins developed using the CellChat v2 database, which provides information on the interactions of ligand–receptor complexes. (F) Venn diagram depicting the 7 exosomal membrane proteins and 621 target proteins. The exosomal membrane proteins are included in the receptor gene set of 74 proteins that were up-regulated in H1975/OR and H1975/WR exosomes compared to H1975 exosome (top box), and 621 target proteins are included in the ligand gene set (bottom box). (G) Circos plot showing the interactions between the 7 exosomal membrane proteins and 75 target proteins reported to be expressed in the cerebral cortex. The red lines in the center represent interactions between proteins, and interacting proteins are marked with stars. (H) LC-MS/MS analysis of exosomal membrane proteins expected to interact in the cerebral cortex. (I) Immunoblot analysis of H1975, H1975/OR, and H1975/WR cells and their exosomes for selected proteins. (J) Five cerebral cortex genes predicted to interact with exosomal membrane proteins. Expression levels of each gene in the cerebral cortex are expressed as nTPM. **P < 0.005 and ***P < 0.0005.

We hypothesized that specific exosomal components, particularly altered protein cargos from EMT-associated resistant cells, enhance exosomal uptake by the brain and facilitate tumor metastasis. To test this hypothesis, we first analyzed the peptide fragmentation patterns of exosomes isolated from the H1975, H1975/OR, and H1975/WR cell lines. Exosomes derived from resistant cells (H1975/OR and H1975/WR) exhibited similar fragmentation profiles, which were distinct from those of the parental H1975 exosomes (Fig. 3A). Subsequently, liquid chromatography-tandem mass spectrometry (LC-MS/MS) proteomic analyses confirmed high reproducibility among biological replicates, as demonstrated by principal components analysis (PCA) (Fig. 3B). LC-MS/MS analysis indicated that more proteins were detected in the parental H1975 exosomes compared to those derive from 2 resistant cells (H1975, 1514 ± 191.6; H1975/OR, 699 ± 66.3; H1975/WR: 636 ± 104.3) (Fig. S3A). Interestingly, although the 2 resistant cells developed resistance to different drugs, the characteristics of the exosomal proteins secreted by both cells were similar on the basis of Gene Ontology (GO) analysis (Fig. S3A). Next, comparative LC-MS/MS identified 744 significantly altered proteins in exosomes derived from both resistant cell lines compared to the parental H1975 exosomes, as visualized using Venn diagrams and heatmaps (fold change ≥ 2; *P* ≤ 0.05) (Fig. 3C and D). GO analyses categorized these proteins into biological process (BP), cellular component (CC), and molecular function (MF), revealing strong enrichment for functions related to cell–cell adhesion and extracellular matrix interactions (Fig. 3E).

Focusing on membrane proteins that are commonly up-regulated in resistant cell-derived exosomes, we constructed an inferred interaction network between exosomal membrane proteins and their potential ligands using the CellChat v2 database. Among the 74 highly expressed exosomal proteins identified through LC-MS/MS, we selected 7 exosomal membrane proteins classified as receptor genes in the CellChat database. Potential ligand candidates were subsequently identified from the Human Protein Atlas (HPA) database, resulting in 621 candidate proteins (609 monomeric and 12 multimeric) (Fig. 3F). These candidates were then stratified according to organ-specific transcriptomic profiles across 40 organs (Fig. S4A), with a particular focus on 4 major metastatic target organs: the brain, liver, lung, and bone marrow. CellChat-based ligand–receptor interaction analyses revealed that among the 7 exosomal membrane proteins, 3 (ITGAV–ITGB3, ITGA2B–ITGB3, and L1CAM) exhibited interactions with 5 brain-specific ligands, strongly suggesting their roles in brain-specific tropism (Fig. 3G). In contrast, 2 exosomal membrane proteins (ITGAV–ITGB3 and ITGA2B–ITGB3) interacted with 4 liver-specific targets, whereas L1CAM showed no significant interactions in the liver, lung, or bone marrow, Additional bioinformatic analysis of 260 commonly enriched genes, identified from the overlap between EMT-resistant cell-specific mRNA sequencing (3,709 genes) and exosomal proteomics (744 proteins), confirmed the presence of L1CAM and ITGB3 (Fig. S5). To experimentally validate these bioinformatics findings, we analyzed the exosomal membrane proteins ITGB3, ITGAV, and L1CAM using LC-MS/MS. ITGB3 and L1CAM were significantly elevated in H1975/OR and H1975/WR exosomes compared to parental H1975 exosomes, whereas ITGAV exhibited inconsistent differences across the groups (Fig. 3H), and ITGA2B was undetectable in all samples. Immunoblot analyses further confirmed the elevated expression of ITGB3 and L1CAM in both cells and exosomes from resistant cell lines, with notably stronger expression observed in exosomes (ITGB3 was highest in H1975/WR; L1CAM was highest in H1975/OR) (Fig. 3I). Finally, the expression levels of 5 brain-specific proteins (NCAM1, contactin 2 [CNTN2], L1CAM, tenascin R [TNR], and NCAM2), predicted to interact with the exosomal membrane proteins ITGB3 and L1CAM, were quantified using normalized transcripts per million (nTPM) from cerebral cortex datasets (Fig. 3J). Collectively, these results suggest that exosomal membrane proteins, such as ITGB3 and L1CAM, interact with cerebral-cortex-specific proteins to promote the BrM of lung cancer.

### Exosomal L1CAM promotes BrM

To determine whether ITGB3 or L1CAM expression is associated with EMT, we treated the human lung cancer cell lines H1975 and A549 with transforming growth factor–β1 (TGF-β1) (5 ng/ml). This treatment resulted in a time-dependent increase in the expression of ITGB3 and L1CAM, consistent with previous reports linking these proteins to EMT processes (Fig. S6) [27,28]. In addition, exosomes from H1975, H1975/OR, and H1975/WR cells were fractionated using iodixanol density gradient ultracentrifugation. Immunoblotting revealed a predominant localization of ITGB3 and L1CAM in fraction 6 (density range, 1.10 to 1.19 g/ml), which corresponds to the typical density of exosomes (Fig. S7). Although these data alone do not clarify whether ITGB3 and L1CAM directly induce EMT or confer resistance to EGFR-TKIs, the results suggest that increased expression of ITGB3 and L1CAM may occur during EMT progression, leading to their secretion via exosomes.

**Fig. 4.**
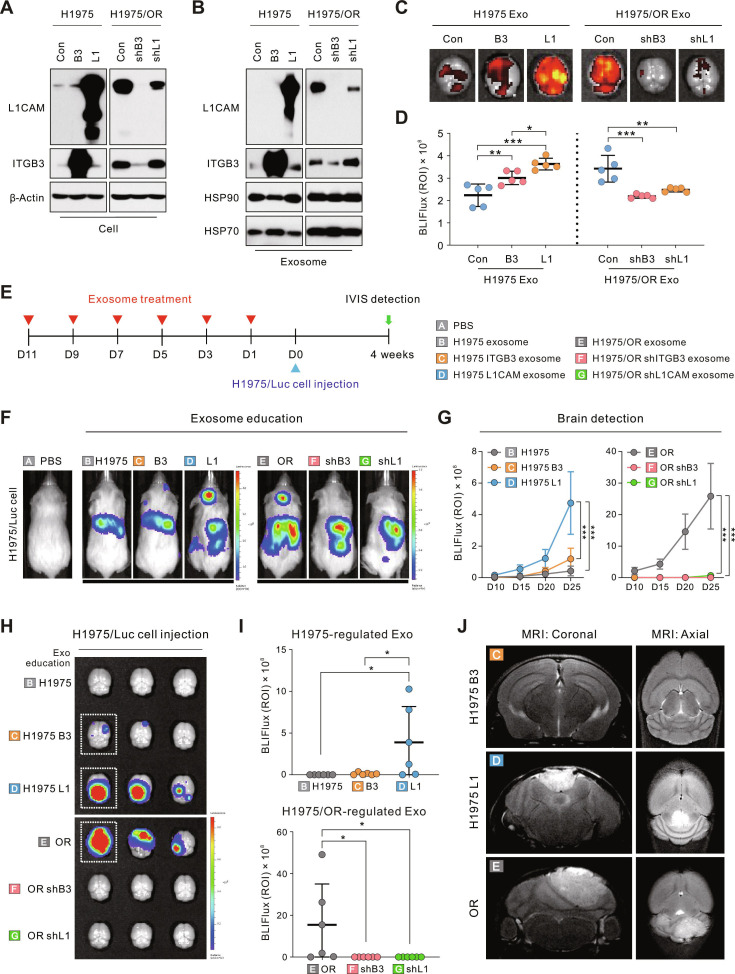
Regulation of BrM through the modulation of exosomal ITGB3 and L1CAM expression. (A and B) Introduction of overexpression (B3 and L1) or knockdown (shB3 and shL1) constructs targeting ITGB3 and L1CAM into cell lines. Overexpression and shRNA empty vectors were used as CONs. After isolating exosomes from the stabilized cell line, protein expression in cells (A) and exosomes (B) was confirmed through immunoblotting. (C) Representative IVIS images of the brain distribution following intracardiac injection of each NIR-labeled exosome. (D) Quantification of exosome biodistribution levels in the brain. (E) Schematic diagram of the exosome education model for metastasis detection. Information regarding PBS and exosomes injected into mice is shown in the legends A to G on the right. (F) Representative IVIS images illustrating the metastatic patterns in mice injected with H1975/Luc cells following each exosome education. (G) Flux values of brain regions at each time point quantified using the IVIS system. (H) Representative IVIS images of mouse brains excised 30 days after intracardiac injection of H1975/Luc cells in an exosome education mouse model. (I) Quantified IVIS values from excised brains. Quantification of ITGB3 and L1CAM overexpressing exosomes compared to H1975 exosomes is shown at the top, whereas the quantification of shITGB3 and shL1CAM compared to H1975/OR exosomes is shown at the bottom. (J) Representative MRI images of brains from each exosome-treated group, including H1975 ITGB3, H1978 L1CAM, and H1975/OR, which exhibited metastatic potential based on IVIS results on the day of sacrifice. Statistical significance of biodistribution levels and BrM is indicated by asterisks. Data are presented as means ± SD (*n* = 5 mice per group). *P < 0.05, **P < 0.005, and ***P < 0.0005.

To directly investigate the role of exosomal ITGB3 and L1CAM in BrM, we generated H1975 cells that overexpress either ITGB3 or L1CAM, as well as H1975/OR cells with short hairpin RNA (shRNA)-mediated knockdown of ITGB3 or L1CAM. Exosomes were isolated from conditioned media, and the changes in expression were confirmed through immunoblot analysis in both cellular and exosomal fractions (Fig. 4A and B). Interestingly, the knockdown of ITGB3 in H1975/OR cells resulted in a concurrent reduction of L1CAM in both the cells and the exosomes (Fig. 4A and B). Further experiments using shRNA targeting ITGB3 consistently demonstrated a decrease in L1CAM expression at both mRNA and protein levels across various cell lines (H1975, H1975/OR, H1975/WR, and HCC827), indicating that ITGB3 signaling may function as an upstream modulator capable of regulating L1CAM transcription (Fig. S8).

Next, we evaluated whether exosomal ITGB3 and L1CAM influence brain uptake in vivo. NIR-labeled exosomes were injected intracardially into mice, and the brains were harvested 24 h postinjection. IVIS imaging revealed a significantly increased accumulation of exosomes in the brains of mice injected with H1975–ITGB3 and H1975–L1CAM cells compared to those receiving H1975–CON exosomes, with the highest uptake observed in the H1975–L1CAM group. Conversely, the brain uptake of exosomes from H1975/OR–shITGB3 and H1975/OR–shL1CAM cells was significantly reduced relative to H1975/OR–CON exosomes (Fig. 4C and D and Fig. S9). On the basis of these findings, we further investigated whether these 2 proteins also directly promote BrM. Thus, NSG mice underwent exosome education, followed by intracardiac injection of H1975/Luc cells. Mice preeducated with H1975–L1CAM exosomes demonstrated significantly enhanced BrM compared to those educated with either H1975–CON or H1975–ITGB3 exosomes. Conversely, the knockdown of either ITGB3 or L1CAM in H1975/OR exosomes significantly reduced brain metastatic lesions compared to H1975/OR–CON exosomes (Fig. 4E to G). Luciferase expression analysis in extracted brains confirmed a markedly higher metastatic burden in groups treated with L1CAM-enriched exosomes (H1975–L1CAM and H1975/OR–CON) (Fig. 4H and I). MRI imaging further validated the presence of distinct metastatic lesions in these groups (Fig. 4J). Collectively, these findings indicate that although exosomal ITGB3 partially contributes to the distribution of exosomes to the brain, exosomal L1CAM plays a major functional role in promoting BrM of lung cancer cells, primarily by facilitating selective exosome uptake into the brain and promoting the establishment of the metastatic niche.

### Exosomal L1CAM mediates BBB permeability by disrupting endothelial junctions and inducing transcriptional remodeling

**Fig. 5.**
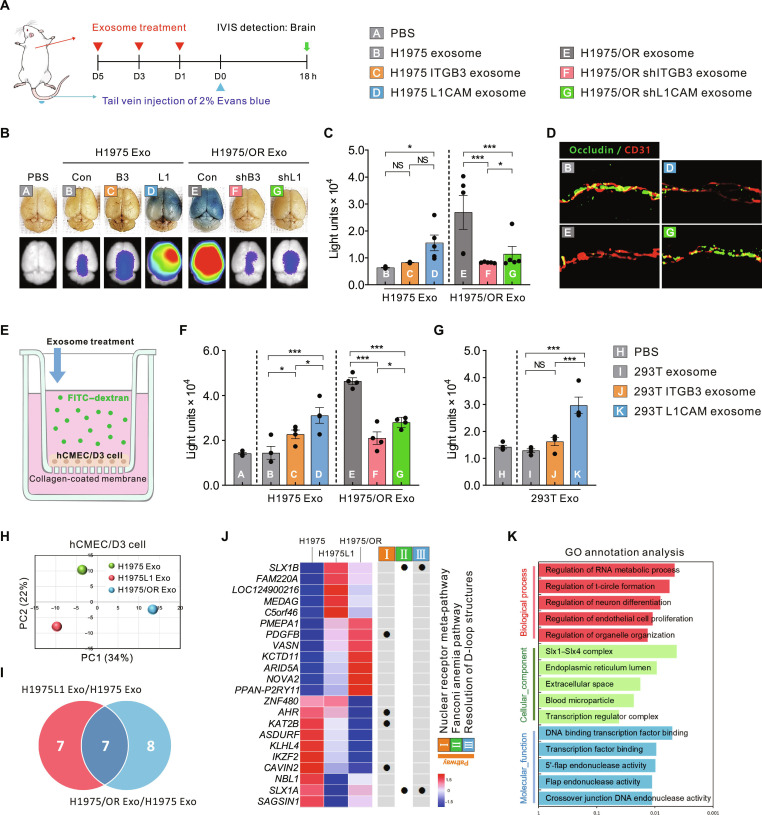
Role of exosomal L1CAM in regulating BBB permeability. (A) Schematic diagram of the Evans blue dye permeability assay model to determine changes in mouse BBB permeability through exosome treatment. Information regarding PBS and exosomes injected into mice is shown in the legend A to K. (B) Representative images of mouse brains excised 18 h after tail vein injection of Evans blue dye. IVIS images showing fluorescence of Evans blue dye are located at the bottom. (C) Quantified IVIS values from the excised brains (*n* = 5 per group). (D) Immunofluorescence detection results showing expression of occludin (green) in excised mouse brain endothelial cells (red) after treatment with exosomes (B, D, E, and G) without Evans blue. (E) Schematic of the in vitro BBB permeability assay. Human BBB cell lines (HCMEC/D3) were cultured as monolayers on collagen-coated membranes within inserts, and changes in permeability after individual exosome treatment were quantified using FITC-conjugated dextran transferred to a receiving tray. (F and G) Quantification of FITC–dextran was used to assess in vitro BBB permeability via H1975 and H1975/OR exosomes B to G (F) and 293T exosomes I to K (G). (H to K) The mRNA sequencing dataset was derived from HCMEC/D3 cells treated with H1975, H1975L1, and H1975/OR exosomes. (H) PCA plot of the merged mRNA sequencing datasets. (I) Venn diagram showing significantly up- and down-regulated mRNA counts (*P* < 0.05) by more than 2-fold between groups. (J) Heatmap showing differentially expressed genes through KEGG analysis. (K) GO term enrichment analysis was based on 22 genes that showed significant increases and decreases in each group. Data are presented as means ± SD. *P < 0.05 and ***P < 0.0005. NS, not significant.

To directly evaluate whether exosomal L1CAM enhances BBB permeability during BrM, we conducted an in vivo permeability assay. Mice were intracardially injected with exosomes 3 times (every 2 days), followed by intravenous administration of Evans blue dye. After 18 h, the extent of Evans blue extravasation into brain tissues was assessed (Fig. 5A). Mice treated with H1975–L1CAM exosomes exhibited significantly increased Evans blue leakage into the brain compared to those in the PBS-, H1975–CON-, and H1975–ITGB3 exosome-treated groups. Conversely, the BBB permeability enhancement induced by H1975/OR exosomes was notably reduced upon treatment with H1975/OR–shITGB3 or H1975/OR–shL1CAM exosomes (Fig. 5B and C). To assess endothelial integrity, we performed immunofluorescence staining for the endothelial marker CD31 and the tight junction protein occludin in mouse brain tissues. Clear disruption of tight junctions was observed in the H1975–L1CAM exosome-treated group, whereas the knockdown of L1CAM in H1975/OR exosomes significantly reduced junctional gap formation (Fig. 5D). These results indicate that exosomal L1CAM directly impairs BBB integrity by targeting endothelial tight junctions.

To further validate exosome-induced permeability in vitro, we performed permeability assays using human cerebral microvascular endothelial cells (HCMEC/D3). HCMEC/D3 cells were cultured on collagen-coated membranes in the upper chambers of Transwell inserts for 72 h to establish stable endothelial monolayers. Each exosome group was then added to the upper chamber, followed by fluorescein isothiocyanate (FITC)–dextran treatment (Fig. 5E). H1975–L1CAM exosomes significantly increased transendothelial permeability, as demonstrated by the translocation of FITC–dextran, compared to the CON and H1975–ITGB3 exosome groups. Conversely, shRNA-mediated suppression of ITGB3 or L1CAM in H1975/OR exosomes reversed these permeability effects (Fig. 5F). Similar results were obtained with 293T–L1CAM-derived exosomes (Fig. 5G). Notably, these permeability changes were not observed in human umbilical vein endothelial cells, suggesting a cell-type-specific response (Fig. S10A and B). Consistent with this brain-endothelial-specific phenotype, supplementary analyses further suggested preferential interaction and functional involvement of brain endothelial surface molecules in exosomal-L1CAM-mediated permeability effects (Fig. S11). Although these differences may be attributed to distinct endothelial characteristics between brain and peripheral endothelium, further analyses are required to elucidate the precise underlying mechanisms.

To elucidate the molecular mechanisms underlying exosome-induced BBB permeability, we conducted mRNA sequencing on HCMEC/D3 cells treated with H1975, H1975–L1CAM, or H1975/OR exosomes. PCA confirmed the reproducibility among replicates (Fig. 5H). Differential gene expression analysis identified 22 genes that were significantly altered following treatment with H1975–L1CAM or H1975/OR exosomes compared with CONs (Fig. 5I and J). Among these, genes such as *PDGFB*, *AHR*, *KAT2B*, and *CAVIN2* have previously been implicated in the regulation of endothelial proliferation, vascular remodeling, and barrier function. Notably, *PDGFB* is a critical regulator of endothelial cell proliferation and vascular integrity [29]. Its up-regulation suggests a role in promoting angiogenesis and maintaining vascular stability. Conversely, *AHR* and *KAT2B* are transcriptional regulators involved in nuclear receptor signaling and chromatin remodeling [30,31]. Their down-regulation may indicate alterations in transcriptional programs affecting endothelial function. As *CAVIN2* is associated with caveolae formation and endothelial barrier function [32], its decreased expression could compromise barrier integrity. Furthermore, altered expression of *SLX1A* and *SLX1B*, components of the Fanconi anemia pathway that play a role in D-loop structure resolution, indicates activation of the DNA damage response and replication stress pathways following exosomal exposure [33] (Fig. 5J and K). Collectively, these transcriptional changes suggest that exosomal L1CAM contributes to BBB disruption by coordinating the disassembly of endothelial junctions and facilitating transcriptional remodeling, thereby establishing a permissive microenvironment that promotes BrM.

### Exosomal ITGB3 and L1CAM serve as effective biomarkers for diagnosing BrM in lung cancer patients

**Fig. 6.**
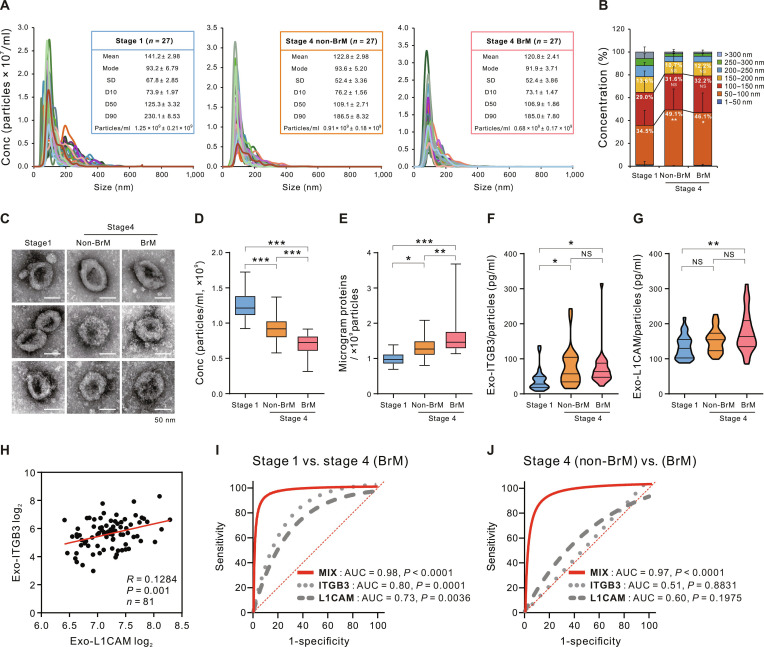
Diagnostic utility of exosomal L1CAM and ITGB3 in patients with lung cancer BrM. Characterization of serum-derived exosomes from patients with stage 1 lung cancer, stage 4 lung cancer (non-BrM), and stage 4 lung cancer (BrM). (A) Size distribution and concentration (Conc) of isolated particles determined by nanoparticle tracking analysis. (B) Stacked bar graph showing the percentage of exosomes by size. (C) Transmission electron microscopy image showing the morphology of patient-derived exosomes. (D) Bar graph showing the number of exosome particles and (E) protein concentration per particle by patient group. (F and G) Expression of the exosomal proteins ITGB3 and L1CAM in each group was measured by ELISA analysis, and the results were normalized to particle number in individual samples. (H) Scatterplot showing the correlation between serum-derived exosomal L1CAM and ITGB3 protein expression in patients with lung cancer. (I and J) ROC analysis of exosomal ITGB3 and L1CAM, individually and in combined sets, for diagnostic efficacy evaluation. (I) ROC analysis of stage 1 and stage 4 (BrM) groups of patients with lung cancer. (J) Stage 4 (non-BrM) and stage 4 (BrM) groups of patients with lung cancer. The ROC curves for the combined sets of exosomal ITGB3 and L1CAM were generated on the basis of the predicted probability for each patient. *P < 0.05, **P < 0.005, and ***P < 0.0005.

To validate the clinical relevance of exosomal ITGB3 and L1CAM in lung-cancer-associated BrM, exosomes from serum samples were isolated, characterized, and divided into 3 groups, each consisting of 27 patients: stage I lung cancer, stage IV lung cancer without BrM (non-BrM), and stage IV lung cancer with BrM (Fig. 6A). The patient groups were comparable in terms of age, sex distribution, EGFR mutation status, and smoking status, with additional detailed clinicopathological characteristics provided in Table 1.

**Table 1.** Clinicopathologic characteristics of patients

Variables	Stage 1	Stage 4	
		Non-BrM	BrM
**Patients, *n***	27	27	27
**Age, years**	64.3 ± 9.1	65.0 ± 8.4	59.8 ± 6.8
**Male sex**	11 (40.7)	15 (55.6)	14 (51.9)
**Smoking status**			
Never	15 (55.6)	16 (59.3)	16 (59.3)
Former/current	12 (44.4)	11 (40.7)	11 (40.7)
Pack/year	31.5 ± 24.2	31.1 ± 22.6	31 ± 13.0
**Underlying comorbidities**			
Respiratory diseases	6 (22.2)	3 (11.1)	3 (11.1)
Cardiovascular diseases	4 (14.8)	13 (48.1)	9 (33.3)
Other medical conditions	11 (40.7)	7 (25.9)	2 (7.4)
**Overall survival, months** (median ± SD)	73.0 ± 18.3	46.9 ± 29.1	33.7 ± 29.8
**Lymph node metastasis**	0 (0)	19 (70.4)	27 (100)
**Distant metastatic site**			
Brain	–	–	27 (100)
Other (without BrM)	–	27 (100)	–
**EGFR single mutations**	21 (77.8)	17 (63.0)	15 (55.6)
Exon 19 deletion	11 (40.7)	8 (29.6)	9 (33.3)
Exon 21 L858R	8 (29.6)	6 (22.2)	4 (14.8)
Other mutation	2 (7.4)	3 (11.1)	2 (7.4)
**Treatment modality**			
CCRT	–	–	–
Chemotherapy	5 (18.5)	27 (100)	27 (100)
**Surgery**	26 (96.3)	3 (11.1)	0 (0)

CCRT, concurrent chemoradiotherapy. Data represent *n* (%) or means ± SD unless stated otherwise.

NanoSight analysis demonstrated that the modal diameter of isolated particles was less than 100 nm, with the majority falling within the standard exosome size range (50 to 200 nm) (Fig. 6B). Notably, the proportion of exosomes measuring between 50 and 100 nm was significantly higher in both stage 4 groups compared to stage 1 (Fig. 6B). Transmission electron microscopy further confirmed the presence of round, membrane-bound vesicles with typical exosomal morphology in representative samples (Fig. 6C). In contrast, total number of particles followed the order: stage 4 BrM < stage 4 non-BrM < stage 1 (Fig. 6D). However, the protein content per particle was elevated in stage 4 patients relative to stage 1, with the highest levels observed in the stage 4 BrM group (Fig. 6E). Exosomal ITGB3 and L1CAM protein levels were quantitatively assed using enzyme-linked immunosorbent assay (ELISA) and normalized to against particle counts. ITGB3 protein expression significantly increased in both the stage 4 non-BrM and stage 4 BrM groups compared to stage 1; however, no significant difference was observed between the stage 4 non-BrM and BrM groups (Fig. 6F).

In contrast, L1CAM expression increased significantly only in the stage 4 BrM group compared with stage 1 (Fig. 6G). Scatter plot analysis of the entire cohort of 81 patients revealed a positive correlation between exosomal ITGB3 and L1CAM protein levels (Fig. 6H). To further support assay robustness, we cross-validated ELISA-based measurements using Western blot analysis in randomly selected patient samples, demonstrating consistent expression patterns across platforms (Fig. S12).

Subsequently, receiver operating characteristic (ROC) curve analysis was performed to assess the diagnostic accuracy of ITGB3 and L1CAM for lung cancer BrM. The combined ITGB3 and L1CAM panel significantly improved diagnostic performance (area under the curve [AUC] = 0.98, *P* < 0.0001) in differentiating stage 4 (BrM) from stage 1, compared to the individual markers ITGB3 (AUC = 0.80, *P* = 0.0001) and L1CAM (AUC = 0.73, *P* = 0.0036) (Fig. 6I). Notably, among patients with stage 4 lung cancer, the combined ITGB3 and L1CAM panel demonstrated the highest discriminative ability (AUC = 0.97, *P* < 0.0001) for distinguishing between non-BrM and BrM subgroups, in stark contrast to the lack of significant diagnostic value for ITGB3 alone (AUC = 0.51, *P* = 0.8831) or L1CAM alone (AUC = 0.60, *P* = 0.1975) (Fig. 6J). These findings were further supported by repeated internal validation using cross-validation and bootstrap resampling, as well as independent machine learning algorithms, which consistently demonstrated superior performance of the combined panel (Fig. S13). In addition, the combined exosomal ITGB3 and L1CAM panel’s efficiency in predicting BrM was unaffected by EGFR mutation status (Fig. S14), although EGFR mutations are strongly associated with a higher risk of BrM in NSCLC [34,35]. Collectively, these findings suggest that the combined exosomal ITGB3 and L1CAM biomarker panel offers excellent diagnostic accuracy for detecting BrM in patients with lung cancer and may have utility as both a diagnostic and prognostic marker for early BrM.

## Discussion

This study identified exosomal L1CAM as a crucial mediator and biomarker of BrM in lung cancer, significantly enhancing our understanding of the mechanisms underlying organotropic metastasis. Specifically, we demonstrate that exosomes derived from EGFR-TKI-resistant lung cancer cells exhibiting EMT characteristics preferentially target the brain microenvironment, thereby facilitating metastatic colonization.

Although L1CAM has previously been recognized as a surface molecule that supports tumor cell adhesion to the brain vasculature [17,21], our findings are the first to reveal its functional role within exosomes in the formation of a premetastatic niche in the brain. This mechanism complements prior studies on exosomal integrins [13] and other organotropic factors [36], offering additional insight into how tumor-derived exosomes contribute to distant niche priming and BrM. A significant strength of our approach is the use of EGFR-TKI-resistant lung adenocarcinoma cell lines, which exhibit enhanced EMT phenotypes. Clinically, patients with EGFR-mutant NSCLC often develop BrM [37], particularly following TKI resistance associated with EMT-like alterations [38–41]. This suggests that therapeutic resistance and metastatic progression may share common molecular pathways that could be exploited as biomarkers or therapeutic targets. Thus, our experimental model enhances the translational relevance of our findings by reflecting clinically pertinent conditions.

Proteomic and transcriptomic analyses revealed elevated levels of L1CAM and ITGB3 in exosomes derived from EMT-resistant cells, consistent with previous reports linking L1CAM to tumor proliferation, migration, and invasion across diverse cancer types [24,42,43]. Moreover, we demonstrated increased expression of both L1CAM and ITGB3 under TGF-β-induced EMT conditions, confirming that this phenomenon also occurs in wild-type EGFR lung cancer cells and is not limited to mutant EGFR-positive adenocarcinomas. Furthermore, knockdown experiments indicated a regulatory relationship between L1CAM and ITGB3, suggesting that ITGB3 signaling may function as an upstream modulator of L1CAM transcription. Supporting this potential association, previous studies have reported concurrent up-regulation of L1CAM and ITGB3 in colorectal cancer, correlating with activation of the extracellular-signal-regulated kinase 1/2 (ERK1/2) pathway and enhanced metastatic capabilities [44]. In addition, an EMT-related gene coexpression network analysis in colorectal cancer further highlighted the prognostic significance of L1CAM and ITGB3 coexpression, reinforcing their functional relevance in metastatic progression [45]. Both L1CAM and ITGB3 share common downstream signaling pathways, including mitogen-activated protein kinase/ERK and phosphatidylinositol 3-kinase/AKT, which are critical for regulating cell migration, invasion, and metastasis [16,24,46]. Although the direct transcriptional regulatory mechanisms between ITGB3 and L1CAM were not fully elucidated in this study, their functional overlap underscores a potential interplay in metastatic progression.

Notably, our study provides strong evidence that exosomal L1CAM independently drives BrM progression. Using various experimental systems, including L1CAM overexpression, knockdown, and exosomes derived from L1CAM-transfected 293T cells, we demonstrated that L1CAM significantly enhances brain-specific biodistribution and increases BBB permeability, thereby establishing a metastatic environment. Although the impact of other exosomal components on metastasis cannot be dismissed, the interaction between exosomal L1CAM and BBB endothelial cells is likely crucial to the metastatic process. Consistent with this notion, supplementary analyses indicate that exosomal L1CAM shows preferential uptake and functional activity in brain endothelial cells, suggesting a brain-endothelium-biased effect (Fig. S11). Notably, disruption of L1CAM–NCAM1-associated interactions in BBB endothelial cells was accompanied by a reduction in BBB permeability. While these findings do not establish a definitive molecular interaction, they suggest that specific endothelial surface molecules may contribute to the brain-selective effects of exosomal L1CAM. Consequently, exosomal L1CAM has significant clinical implications as a targeted delivery vehicle for brain-related applications.

Clinically, we developed a combined biomarker panel that integrates exosomal L1CAM and ITGB3, based on their functional complementarity and positive correlation. Our panel demonstrated superior diagnostic accuracy in identifying patients with lung cancer and BrM compared to either marker alone. Although previous biomarkers, such as exosomal integrin α_v_β_3_ [13], cell-migration-inducing hyaluronidase 1 (CEMIP) [36], and miR-181c [47] have shown potential, their clinical validation remains limited. In contrast, our panel significantly enhances sensitivity and specificity, providing an adequate, noninvasive liquid biopsy for the early detection and monitoring of BrM in clinical practice.

Despite these strengths, our study has several limitations. First, our analysis primarily focused on endothelial cell interactions; therefore, the potential roles of other brain-resident cells, such as astrocytes and microglia, warrant further investigation. Second, detailed molecular biological analysis of the formation of metastatic niches following exosome delivery to the brain was lacking, underscoring the need for further research. Third, prospective longitudinal studies are necessary to validate the clinical utility of our biomarker panel. Finally, therapeutic validation of L1CAM-targeted strategies in preclinical models is essential; however, a significant limitation is the lack of animal models that spontaneously develop BrM from primary lung cancer.

In summary, our findings highlight exosomal L1CAM as a critical regulator of BrM, facilitating brain-specific biodistribution and BBB disruption to establish a premetastatic niche. Furthermore, the combined exosomal L1CAM/ITGB3 panel serves as a promising clinical biomarker for risk stratification and early detection of BrM in patients with lung cancer.

## Materials and Methods

### Establishment of cells resistant to third-generation EGFR-TKIs

Human NSCLC cell line H1975 with the EGFR L858R/T790M double mutation was purchased from the American Type Culture Collection (Rockville, MD, USA, RRID:CVCL_1511). The cells were cultured in RPMI 1640 medium (Invitrogen, Carlsbad, CA, USA) supplemented with 10% fetal bovine serum, penicillin (100 U/ml), and streptomycin (100 mg/ml; Invitrogen, Carlsbad, CA) at 37 °C in an atmosphere with 5% CO_2_. H1975/OR and H1975/WR cell lines with acquired resistance to third-generation EGFR-TKIs (osimertinib and WZ4002) were established from the H1975 cells. To eliminate the effects of each drug, the resistant cell lines (H1975/OR and H1975/WR) were cultured in drug-free medium for at least 1 week before experimentation. The resistant cell lines were authenticated using short tandem repeat analysis and confirmed to be mycoplasma-free using standard methods. The detailed methodology for establishing the resistant cell lines was described in a previous paper [26].

### Mouse study

All mice were maintained under specific pathogen-free conditions in the Department of Laboratory Animal Research at Medical Center. All animal procedures were approved and conducted in accordance with guidelines established by the Korean Ministry of Food and Drug Safety.

Female NSG mice (18 to 20 g, 6 weeks old, RRID:IMSR_JAX:005557) were used for exosome education and metastasis assays. For the exosome education experiments, exosomes (2 × 10^9^ particles) resuspended in 100 μl of PBS were intracardially injected into the mice every other day for a total of 6 injections prior to tumor cell injection. Luciferase-expressing tumor cell lines (H1975, H1975/OR, and H1975/WR) were generated by infection with RediFect Red-Fluc-Puro lentiviral particles (#CLS960002, PerkinElmer, Norwalk, CT, USA) and selected using puromycin. Luciferase-expressing cells (0.5 × 10^6^ cells/100 μl of PBS) were injected intracardially into the mice using a 31-gauge insulin syringe. Tumor burden and metastatic dissemination were monitored twice per week using an IVIS imaging system (PerkinElmer, RRID:SCR_025239) after intraperitoneal injection of d-luciferin potassium salt (150 mg/kg; PerkinElmer). Female C57BL/6 (B6) mice (18 to 20 g, 6 weeks old, RRID:MGI:2159769) were used for exosome biodistribution and BBB permeability analyses. The descriptions of each experiment are provided separately.

### Transcriptomic and proteomic profiling

Transcriptomic (mRNA sequencing) and proteomic (LC-MS/MS) analyses were performed on the H1975, H1975/OR, and H1975/WR cell lines and their corresponding secreted exosomes by Ebiogen Inc. (Seoul, Korea). For proteomic profiling, exosomal protein concentrations were determined using the Pierce BCA Protein Assay Kit (Thermo Fisher Scientific, Waltham, MA, USA). The proteins were reduced, digested, and desalted using the filter-aided sample preparation method with Microcon 30K centrifugal filter units (Millipore, Billerica, MA, USA). Peptides were analyzed using an UltiMate 3000 RSLC nano-LC system (Thermo Fisher Scientific, RRID:SCR_026145) coupled with a Q Exactive mass spectrometer (Thermo Fisher Scientific). Raw MS/MS spectra were converted to mzXML format via MSConvert and searched using the Andromeda engine within MaxQuant (v1.5.8.3, RRID:SCR_014485). Peptide and protein identifications were based on mass-to-charge ratio, retention time, and peak intensities. Datasets were filtered using EXDEGA software (Ebiogen) to retain proteins with >10% sequence coverage for downstream bioinformatic analysis.

For transcriptome profiling, total RNA was extracted from cells, and libraries were prepared using the NEBNext Ultra II Directional RNA-Seq Kit (New England Biolabs, UK). Polyadenylated mRNA was isolated using the Poly(A) (polyadenylate) RNA Selection Kit (Lexogen, Austria), followed by cDNA synthesis and fragmentation. Indexed libraries (Illumina indexes 1 to 12) were polymerase chain reaction (PCR)-amplified and assessed for average fragment size using the TapeStation HS D1000 Screen Tape (Agilent Technologies, The Netherlands). Quantification was performed using the StepOne Real-Time PCR System (Life Technologies, USA), and sequencing was conducted on the Illumina NovaSeq 6000 platform with paired-end 100-bp reads. Raw sequencing data were quality-checked using FastQC (RRID:SCR_014583), and adapter sequences or low-quality reads (<Q20) were removed using FASTX_Trimmer and BBMap (RRID:SCR_016965). Clean reads were aligned to the human reference genome with TopHat (RRID:SCR_013035). Gene expression values were normalized using the FPKM+Geometric normalization method implemented in EdgeR (RRID:SCR_012802) within the R environment, and fragments per kilobase per million mapped reads (FPKM) values were estimated using Cufflinks (RRID:SCR_014597).

### Bioinformatics and ligand–receptor interaction analysis

Proteomic data from exosomes derived from EMT-associated resistant cells were analyzed using the MaxQuant software package (v1.6.6.0). Secondary MS spectra were searched against the Swiss-Prot Human database (Proteome ID: UP000005640; 20,600 proteins). GO annotations for BPs, CCs, and MFs were assigned using InterProScan (v5.14-53.0, RRID:SCR_005829). Pathway enrichment analysis of differentially expressed proteins was conducted using the Kyoto Encyclopedia of Genes and Genomes (KEGG) database (RRID:SCR_001120) through the KAAS tool (v2.0). To identify exosomal membrane proteins with potential roles in organotropic metastasis, significantly up-regulated proteins from LC-MS/MS were filtered using the DAVID functional annotation tool (RRID:SCR_001881) and annotated for membrane localization (Fig. S5H).

To investigate brain-specific interactions, we analyzed single-cell RNA sequencing expression data from the HPA (https://www.proteinatlas.org/, RRID:SCR_006710) [48]. From the brain tissue dataset, we initially selected 2,658 genes with elevated expression, of which 233 were annotated as plasma membrane proteins. Among these, we prioritized and plotted 16 genes relevant to cancer (Fig. S5I). To explore physical interactions between exosomal proteins (*n* = 38) and brain-enriched membrane proteins (*n* = 16), we used the STRING database (v12.0; https://string-db.org/, RRID:SCR_005223) (Fig. S5J). We constructed protein–protein interaction networks based on experimental evidence and database-derived associations, applying a minimum interaction score threshold of 0.4 (medium confidence). The interaction networks were visualized based on combined STRING scores.

Ligand–receptor interaction analysis was performed by intersecting 74 highly expressed exosomal genes with 780 receptor genes from CellChatDB (v2) [49], resulting in the identification of 7 receptor candidates. Ligand candidates were extracted from the HPA dataset, which includes 805,640 observations across 40 human tissues and 20,141 genes. Genes with nTPM < 1 were excluded, resulting in 18,747 genes. These genes were then matched to the ligand annotations in CellChatDB, yielding 609 monomeric ligands and 12 multimeric ligands. For the multimeric ligands, gene-level expression data were aggregated. Tissue specificity was determined by selecting ligands with maximal expression in a single tissue type. Finally, 39 known ligand–receptor pairs involving the identified receptors were visualized using the circlize R package (v0.4.16, RRID:SCR_002141) [50], focusing on interactions that were enriched in the cerebral cortex, liver, and bone marrow (Fig. 3G and Fig. S4A and B).

### Statistical analysis

Categorical variables were compared using Pearson’s chi-square or Fisher’s exact tests, as appropriate. Continuous variables were analyzed using the Student’s *t* test or 2-way analysis of variance (ANOVA). All statistical tests were 2-sided, and a *P* < 0.05 was considered statistically significant. Statistical analyses were performed using GraphPad Prism software (version 8.0, GraphPad Software Inc., CA, USA, RRID:SCR_002798).

Further detailed methods are provided in the Supplementary Material.

## Data Availability

All data associated with this study are present in the paper or the Supplementary Materials.

## References

[1] Nishino M, Soejima K, Mitsudomi T. Brain metastases in oncogene-driven non-small cell lung cancer. Transl Lung Cancer Res. 2019;8(Suppl 3):S298–S307. 31857953 10.21037/tlcr.2019.05.15 PMC6894990

[2] Barnholtz-Sloan JS, Sloan AE, Davis FG, Vigneau FD, Lai P, Sawaya RE. Incidence proportions of brain metastases in patients diagnosed (1973 to 2001) in the Metropolitan Detroit Cancer Surveillance System. J Clin Oncol. 2004;22(14):2865–2872. 15254054 10.1200/JCO.2004.12.149

[3] Schouten LJ, Rutten J, Huveneers HA, Twijnstra A. Incidence of brain metastases in a cohort of patients with carcinoma of the breast, colon, kidney, and lung and melanoma. Cancer. 2002;94(10):2698–2705. 12173339 10.1002/cncr.10541

[4] Peters S, Bexelius C, Munk V, Leighl N. The impact of brain metastasis on quality of life, resource utilization and survival in patients with non-small-cell lung cancer. Cancer Treat Rev. 2016;45:139–162. 27019457 10.1016/j.ctrv.2016.03.009

[5] Boire A, Brastianos PK, Garzia L, Valiente M. Brain metastasis. Nat Rev Cancer. 2020;20(1):4–11. 31780784 10.1038/s41568-019-0220-y

[6] Kaplan RN, Riba RD, Zacharoulis S, Bramley AH, Vincent L, Costa C, MacDonald DD, Jin DK, Shido K, Kerns SA, et al. VEGFR1-positive haematopoietic bone marrow progenitors initiate the pre-metastatic niche. Nature. 2005;438(7069):820–827. 16341007 10.1038/nature04186 PMC2945882

[7] Colombo M, Raposo G, Thery C. Biogenesis, secretion, and intercellular interactions of exosomes and other extracellular vesicles. Annu Rev Cell Dev Biol. 2014;30:255–289. 25288114 10.1146/annurev-cellbio-101512-122326

[8] Kalluri R, LeBleu VS. The biology, function, and biomedical applications of exosomes. Science. 2020;367(6478): Article eaau6977. 32029601 10.1126/science.aau6977 PMC7717626

[9] Pegtel DM, Gould SJ. Exosomes. Annu Rev Biochem. 2019;88:487–514. 31220978 10.1146/annurev-biochem-013118-111902

[10] Kim DH, Kim H, Choi YJ, Kim SY, Lee JE, Sung KJ, Sung YH, Pack CG, Jung MK, Han B, et al. Exosomal PD-L1 promotes tumor growth through immune escape in non-small cell lung cancer. Exp Mol Med. 2019;51(8):1–13. 10.1038/s12276-019-0295-2 PMC6802663 31399559

[11] Kim DH, Park S, Kim H, Choi YJ, Kim SY, Sung KJ, Sung YH, Choi CM, Yun M, Yi YS, et al. Tumor-derived exosomal miR-619-5p promotes tumor angiogenesis and metastasis through the inhibition of RCAN1.4. Cancer Lett. 2020;475:2–13. 32004570 10.1016/j.canlet.2020.01.023

[12] Kim DH, Park H, Choi YJ, Kang MH, Kim TK, Pack CG, Choi CM, Lee JC, Rho JK. Exosomal miR-1260b derived from non-small cell lung cancer promotes tumor metastasis through the inhibition of HIPK2. Cell Death Dis. 2021;12(8):747. 34321461 10.1038/s41419-021-04024-9 PMC8319168

[13] Hoshino A, Costa-Silva B, Shen TL, Rodrigues G, Hashimoto A, Tesic Mark M, Molina H, Kohsaka S, Di Giannatale A, Ceder S, et al. Tumour exosome integrins determine organotropic metastasis. Nature. 2015;527(7578):329–335. 26524530 10.1038/nature15756 PMC4788391

[14] Srinivasan ES, Tan AC, Anders CK, Pendergast AM, Sipkins DA, Ashley DM, Fecci PE, Khasraw M. Salting the soil: Targeting the microenvironment of brain metastases. Mol Cancer Ther. 2021;20(3):455–466. 33402399 10.1158/1535-7163.MCT-20-0579 PMC8041238

[15] Chen GY, Cheng JC, Chen YF, Yang JC, Hsu FM. Circulating exosomal integrin β3 is associated with intracranial failure and survival in lung cancer patients receiving cranial irradiation for brain metastases: A prospective observational study. Cancers. 2021;13(3):380. 33498505 10.3390/cancers13030380 PMC7864205

[16] Kiefel H, Bondong S, Hazin J, Ridinger J, Schirmer U, Riedle S, Altevogt P. L1CAM: A major driver for tumor cell invasion and motility. Cell Adhes Migr. 2012;6(4):374–384. 10.4161/cam.20832 PMC3478260 22796939

[17] Valiente M, Obenauf AC, Jin X, Chen Q, Zhang XH, Lee DJ, Chaft JE, Kris MG, Huse JT, Brogi E, et al. Serpins promote cancer cell survival and vascular co-option in brain metastasis. Cell. 2014;156(5):1002–1016. 24581498 10.1016/j.cell.2014.01.040 PMC3988473

[18] Maten MV, Reijnen C, Pijnenborg JMA, Zegers MM. L1 cell adhesion molecule in cancer, a systematic review on domain-specific functions. Int J Mol Sci. 2019;20(17):4180. 31455004 10.3390/ijms20174180 PMC6747497

[19] Brummendorf T, Kenwrick S, Rathjen FG. Neural cell recognition molecule L1: From cell biology to human hereditary brain malformations. Curr Opin Neurobiol. 1998;8(1):87–97. 9568396 10.1016/s0959-4388(98)80012-3

[20] Colombo F, Meldolesi J. L1-CAM and N-CAM: From adhesion proteins to pharmacological targets. Trends Pharmacol Sci. 2015;36(11):769–781. 26478212 10.1016/j.tips.2015.08.004

[21] Er EE, Valiente M, Ganesh K, Zou Y, Agrawal S, Hu J, Griscom B, Rosenblum M, Boire A, Brogi E, et al. Pericyte-like spreading by disseminated cancer cells activates YAP and MRTF for metastatic colonization. Nat Cell Biol. 2018;20(8):966–978. 30038252 10.1038/s41556-018-0138-8 PMC6467203

[22] Kiefel H, Bondong S, Erbe-Hoffmann N, Hazin J, Riedle S, Wolf J, Pfeifer M, Arlt A, Schafer H, Müerköster SS, et al. L1CAM-integrin interaction induces constitutive NF-κB activation in pancreatic adenocarcinoma cells by enhancing IL-1β expression. Oncogene. 2010;29(34):4766–4778. 20543863 10.1038/onc.2010.230

[23] Sokeland G, Schumacher U. The functional role of integrins during intra- and extravasation within the metastatic cascade. Mol Cancer. 2019;18(1):12. 30657059 10.1186/s12943-018-0937-3 PMC6337777

[24] Ganesh K, Basnet H, Kaygusuz Y, Laughney AM, He L, Sharma R, O’Rourke KP, Reuter VP, Huang YH, Turkekul M, et al. L1CAM defines the regenerative origin of metastasis-initiating cells in colorectal cancer. Nat Cancer. 2020;1(1):28–45. 32656539 10.1038/s43018-019-0006-x PMC7351134

[25] Wang J-W, Wang H-L, Liu Q, Hu K, Yuan Q, Huang S-K, Wan J-H. L1CAM expression in either metastatic brain lesion or peripheral blood is correlated with peripheral platelet count in patients with brain metastases from lung cancer. Front Oncol. 2022;12: Article 990762. 36387224 10.3389/fonc.2022.990762 PMC9647166

[26] Ji W, Choi YJ, Kang MH, Sung KJ, Kim DH, Jung S, Choi CM, Lee JC, Rho JK. Efficacy of the CDK7 inhibitor on EMT-associated resistance to 3rd generation EGFR-TKIs in non-small cell lung cancer cell lines. Cells. 2020;9(12):2596. 33287368 10.3390/cells9122596 PMC7761809

[27] Kiefel H, Bondong S, Pfeifer M, Schirmer U, Erbe-Hoffmann N, Schafer H, Sebens S, Altevogt P. EMT-associated up-regulation of L1CAM provides insights into L1CAM-mediated integrin signalling and NF-κB activation. Carcinogenesis. 2012;33(10):1919–1929. 22764136 10.1093/carcin/bgs220

[28] Galliher AJ, Schiemann WP. β_3_integrin and Src facilitate transforming growth factor-β mediated induction of epithelial-mesenchymal transition in mammary epithelial cells. Breast Cancer Res. 2006;8(4):R42. 16859511 10.1186/bcr1524 PMC1779461

[29] Andrae J, Gallini R, Betsholtz C. Role of platelet-derived growth factors in physiology and medicine. Genes Dev. 2008;22(10):1276–1312. 18483217 10.1101/gad.1653708 PMC2732412

[30] Sondermann NC, Fassbender S, Hartung F, Hatala AM, Rolfes KM, Vogel CFA, Haarmann-Stemmann T. Functions of the aryl hydrocarbon receptor (AHR) beyond the canonical AHR/ARNT signaling pathway. Biochem Pharmacol. 2023;208: Article 115371. 36528068 10.1016/j.bcp.2022.115371 PMC9884176

[31] Yao Y, Niu Y, Zhou H, Yong M. KAT2B inhibits proliferation and invasion via inactivating TGF-β/Smad3 pathway-medicated autophagy and EMT in epithelial ovarian cancer. Sci Rep. 2025;15(1):3417. 39870682 10.1038/s41598-024-83977-1 PMC11772695

[32] Boopathy GTK, Kulkarni M, Ho SY, Boey A, Chua EWM, Barathi VA, Carney TJ, Wang X, Hong W. Cavin-2 regulates the activity and stability of endothelial nitric-oxide synthase (eNOS) in angiogenesis. J Biol Chem. 2017;292(43):17760–17776. 28912276 10.1074/jbc.M117.794743 PMC5663877

[33] Gaur V, Wyatt HDM, Komorowska W, Szczepanowski RH, de Sanctis D, Gorecka KM, West SC, Nowotny M. Structural and mechanistic analysis of the Slx1-Slx4 endonuclease. Cell Rep. 2015;10(9):1467–1476. 25753413 10.1016/j.celrep.2015.02.019 PMC4407285

[34] Zhao W, Zhou W, Rong L, Sun M, Lin X, Wang L, Wang S, Wang Y, Hui Z. Epidermal growth factor receptor mutations and brain metastases in non-small cell lung cancer. Front Oncol. 2022;12: Article 912505. 36457515 10.3389/fonc.2022.912505 PMC9707620

[35] Ouyang W, Yu J, Zhou Y, Hu J, Huang Z, Zhang J, Xie C. Risk factors of metachronous brain metastasis in patients with EGFR-mutated advanced non-small cell lung cancer. BMC Cancer. 2020;20(1):699. 32723319 10.1186/s12885-020-07202-8 PMC7390194

[36] Rodrigues G, Hoshino A, Kenific CM, Matei IR, Steiner L, Freitas D, Kim HS, Oxley PR, Scandariato I, Casanova-Salas I, et al. Tumour exosomal CEMIP protein promotes cancer cell colonization in brain metastasis. Nat Cell Biol. 2019;21(11):1403–1412. 31685984 10.1038/s41556-019-0404-4 PMC7354005

[37] Kelly WJ, Shah NJ, Subramaniam DS. Management of brain metastases in epidermal growth factor receptor mutant non-small-cell lung cancer. Front Oncol. 2018;8:208. 30018881 10.3389/fonc.2018.00208 PMC6037690

[38] Adua SJ, Arnal-Estape A, Zhao M, Qi B, Liu ZZ, Kravitz C, Hulme H, Strittmatter N, Lopez-Giraldez F, Chande S, et al. Brain metastatic outgrowth and osimertinib resistance are potentiated by RhoA in EGFR-mutant lung cancer. Nat Commun. 2022;13(1):7690. 36509758 10.1038/s41467-022-34889-z PMC9744876

[39] Omuro AM, Kris MG, Miller VA, Franceschi E, Shah N, Milton DT, Abrey LE. High incidence of disease recurrence in the brain and leptomeninges in patients with nonsmall cell lung carcinoma after response to gefitinib. Cancer. 2005;103(11):2344–2348. 15844174 10.1002/cncr.21033

[40] Lee YJ, Choi HJ, Kim SK, Chang J, Moon JW, Park IK, Kim JH, Cho BC. Frequent central nervous system failure after clinical benefit with epidermal growth factor receptor tyrosine kinase inhibitors in Korean patients with nonsmall-cell lung cancer. Cancer. 2010;116(5):1336–1343. 20066717 10.1002/cncr.24877

[41] Lee JS, Hong JH, Sun S, Won HS, Kim YH, Ahn MS, Kang SY, Lee HW, Ko YH. The impact of systemic treatment on brain metastasis in patients with non-small-cell lung cancer: A retrospective nationwide population-based cohort study. Sci Rep. 2019;9(1):18689. 31822734 10.1038/s41598-019-55150-6 PMC6904708

[42] Chen DL, Zeng ZL, Yang J, Ren C, Wang DS, Wu WJ, Xu RH. L1cam promotes tumor progression and metastasis and is an independent unfavorable prognostic factor in gastric cancer. J Hematol Oncol. 2013;6:43. 23806079 10.1186/1756-8722-6-43 PMC3717076

[43] Doberstein K, Spivak R, Reavis HD, Hooda J, Feng Y, Kroeger PT Jr, Stuckelberger S, Mills GB, Devins KM, Schwartz LE, et al. L1CAM is required for early dissemination of fallopian tube carcinoma precursors to the ovary. Commun Biol. 2022;5(1):1362. 36509990 10.1038/s42003-022-04314-8 PMC9744873

[44] Fang QX, Zheng XC, Zhao HJ. L1CAM is involved in lymph node metastasis via ERK1/2 signaling in colorectal cancer. Am J Transl Res. 2020;12(3):837–846. 32269716 PMC7137048

[45] Zhang L, Qian Y. An epithelial-mesenchymal transition-related prognostic model for colorectal cancer based on weighted gene co-expression network analysis. J Int Med Res. 2022;50(12):3000605221140683. 36510452 10.1177/03000605221140683 PMC9751178

[46] Hong SK, Park JR, Kwon OS, Kim KT, Bae GY, Cha HJ. Induction of integrin β3 by sustained ERK activity promotes the invasiveness of TGFβ-induced mesenchymal tumor cells. Cancer Lett. 2016;376(2):339–346. 27085460 10.1016/j.canlet.2016.04.012

[47] Tominaga N, Kosaka N, Ono M, Katsuda T, Yoshioka Y, Tamura K, Lotvall J, Nakagama H, Ochiya T. Brain metastatic cancer cells release microRNA-181c-containing extracellular vesicles capable of destructing blood–brain barrier. Nat Commun. 2015;6:6716. 25828099 10.1038/ncomms7716 PMC4396394

[48] Uhlén M, Fagerberg L, Hallström BM, Lindskog C, Oksvold P, Mardinoglu Å, Sivertsson A, Kampf C, Sjöstedt E, Asplund A, et al. Tissue-based map of the human proteome. Science. 2015;347(6220):1260419. 25613900 10.1126/science.1260419

[49] Jin S, Plikus MV, Nie Q. CellChat for systematic analysis of cell-cell communication from single-cell transcriptomics. Nat Protoc. 2025;20(1):180–219. 39289562 10.1038/s41596-024-01045-4

[50] Gu Z, Gu L, Eils R, Schlesner M, Brors B. *circlize* implements and enhances circular visualization in R. Bioinformatics. 2014;30(19):2811–2812. 24930139 10.1093/bioinformatics/btu393

