# Supplementary material for: Tumor Exosomal L1 Cell Adhesion Molecule Promotes Brain Metastasis of Lung Cancer
Source: Research (Wash D C). 2026 Feb 10;9:1126. doi: 10.34133/research.1126 (PMC12886715; doi:10.34133/research.1126)
Supplement: Supplementary 1 — Materials and Methods Figs. S1 to S15 [file research.1126.f1.pdf]

SUPPLEMENTARY MATERIALS

Tumour Exosomal L1 Cell Adhesion Molecule Promotes Brain Metastasis of Lung Cancer

Dong Ha Kim[1], Chae Won Lee[1], Yun Jung Choi[1], Da-Som Kim[2], Kyosun Ban[2], Juhyeon Hong[2], Gyeong Joon Moon[3], Sang-Yeob Kim[4], Chan-Gi Pack[4], In-Jeoung Baek[5], Jin-Yong Jeong[4], Dong-Cheol Woo[6], Ji-Hye Oh[7], Chang Ohk Sung[7], Kyunggon Kim[8], Hyun-Yi Kim[9], Hae-Yun Jung[10], Wonjun Ji[11], Min Jee Kim[11], Chang Min Choi[11], Jae Cheol Lee[12*], Jin Kyung Rho[2,4*]

*Address correspondence to:

Jae Cheol Lee, MD, PhD

Department of Oncology

Asan Medical Center, University of Ulsan College of Medicine

88, Olympic-ro 43-gil, Songpa-gu, Seoul 05505, South Korea

Tel: +82-2-3010-3208; Fax: +82-2-3010-6961

E-mail: jclee@amc.seoul.kr

and

Jin Kyung Rho, PhD

Department of Convergence Medicine

Asan Medical Center, University of Ulsan College of Medicine

88, Olympic-ro 43-gil, Songpa-gu, Seoul 05505, South Korea

Tel: +82-2-3010-2974; Fax: +82-2-3010-6961

E-mail: jkrho@amc.seoul.kr

MATERIALS AND METHODS

Exosome isolation

From cell culture supernatant using tangential flow filtration

Cells were seeded in 150 mm dishes and cultured until they reached 60%–70% confluency. The following day, the cells were washed twice with and incubated in serum-free medium for 30–48 hours. At the time of harvest, cell confluence exceeded 80%, and cell viability was greater than 90%. The conditioned medium was collected and centrifuged at 2,000 × g for 10 min at 4 °C to remove cellular debris. The supernatant was then filtered through a 0.2 μm syringe filter and concentrated using the Minimate™ EVO Tangential Flow Filtration System (Pall Corporation, Port Washington, NY, USA), which was equipped with a 100k Omega™ membrane capsule (Cat# OA100C12). The operating parameters included a recirculation flow rate of 150–180 rpm and a vacuum pressure of 1.0–1.5 bar. The retentate was collected, quantified using nanoparticle tracking analysis (NTA) and the BCA protein assay (Bio-Rad Laboratories, Hercules, CA, USA), and stored at −80 °C until further use.

From serum samples using ultracentrifugation

For plasma-derived exosomes, serum samples were thawed on ice and centrifuged at 10,000 × g for 30 min at 4 °C to remove residual cells and debris. The supernatant was ultracentrifuged at 100,000 × g for 70 min at 4 °C. The resulting pellet was washed with PBS and subjected to a second ultracentrifugation step under identical conditions. Exosome pellets were resuspended in PBS and quantified by particle count and protein concentration before storage at −80 °C.

Iodixanol density gradient ultracentrifugation (Optional for Purification)

For further purification, 0.5 mL of exosome suspension in PBS was carefully layered onto a discontinuous iodixanol gradient consisting of 3 mL of 40%, 3 mL of 20%, and 3 mL of 10% iodixanol in a 13 mL ultra-clear tube (Beckman Coulter). The gradients were ultracentrifuged at 100,000 × g for 18 hours at 4 °C. Fractions (1.0 mL each) were collected sequentially from

the top of the gradient, and selected exosome-rich fractions were pooled, precipitated with 100% trichloroacetic acid, and washed with 80% ethanol. The dried pellets were resuspended in PBS for downstream analyses or solubilized in RIPA buffer for immunoblotting (Fig. S7).

Nanoparticle tracking analysis

Size distribution and concentration of serum-derived exosomes were analyzed using the NanoSight NS300 system (Malvern Instruments Ltd, Malvern, UK), which leverages light scattering and Brownian motion properties. Isolated exosomes were diluted 100-fold in PBS to reduce the number of visible particles per frame to fewer than 100. This process was repeated three times for 60 seconds at room temperature and 10 frames per second. The results obtained from these repetitions were analyzed using an NTA software (version 2.3 build 0017, RRID:SCR_014239).

Negative staining electron microscopy

Purified exosomes were fixed in 2% (vol/vol) paraformaldehyde for 5 min at room temperature. Following fixation, 10 μg of the exosome suspension was applied to a foam-bar/carbon-coated grid (200 mesh) for 3 min and stained with 2% uranyl acetate. After removing the excess uranyl acetate with filter paper, the grid was examined using a transmission electron microscope (TEM, Hitachi H7600, Japan) at 80 kV.

Histological analysis

Mouse liver, lung, bone, and brain tissues were fixed in 10% buffered formalin, paraffin-embedded, sectioned at 4 μm thickness, and stained with hematoxylin and eosin or Masson's trichrome. Whole-slide images were acquired using a Vectra Polaris Automated Quantitative Pathology Imaging System (Akoya Biosciences, RRID:SCR_025508) at 20× magnification.

MRI of brain metastases

MRI experiments were conducted using a 7 T horizontal-bore PharmaScan 70/16 scanner (Bruker BioSpin GmbH, Ettlingen, Germany) equipped with a 400 mT/m self-shielding gradient system and an actively decoupled cross-coil setup, which consisted of a 72 mm body coil for excitation and a 25 mm single-loop surface coil for mouse brain imaging. During the MRI scans, the animals were anesthetized with medical air (1.0 L/min) containing isoflurane (1.5%–3.0% for induction and 2.0% for maintenance) in a mixture of 70% N_2O and 30% O_2 administered through a nose cone. The animals were immobilized with ear bars during the scanning process. Body temperature was maintained at approximately 37 °C using a warm-water circulation system integrated into a flatbed around the mouse's body. Physiological parameters, including heart and respiratory rates, were monitored using an animal respiratory gating system (SA Instruments, Stony Brook, NY, USA).

Exosome labeling and biodistribution analysis

Exosome labeling was performed using the ExoGlow™-Vivo EV Labeling Kit (Near IR; EXOGV900A-1, System Biosciences, USA) in accordance with the manufacturer's guidelines. After staining, the labeled exosomes were isolated using the Exoquick exosome precipitation solution (System Biosciences, Palo Alto, USA) to remove excess dye and were then resuspended in PBS buffer for *in vivo* imaging. Labeled exosomes (2×10^9 particles/100 µL) were administered systemically via intracardiac injection. To minimize autofluorescence, more than 700 µL of blood was withdrawn through cardiac puncture 24 hours after exosome treatment, and the distribution of exosomes in each organ was confirmed using the near-infrared range (excitation 710 nm, emission 760 nm) of the *In Vivo* Imaging System (IVIS) system (PerkinElmer, RRID:SCR_025239).

Generation of stable knockdown and overexpression cell lines

Lentiviral vectors targeting ITGB3 (NM_000212) and L1CAM (NM_000425) were constructed to establish stable knockdown and overexpression models. For overexpression, an ITGB3 expression plasmid (Origene, RC221606L3, Rockville, MD, USA) and an L1CAM expression plasmid (Origene, RC211601L4) were cloned into a lentiviral backbone. For knockdown studies, shRNA constructs targeting ITGB3 (Sigma-Aldrich, SHCLNG-TRCN0000003235, TRCN0000003234) or L1CAM (Sigma-Aldrich, SHCLNG-TRCN0000063916, TRCN0000063917) were similarly cloned into lentiviral vectors. A scrambled shRNA sequence was used as a negative control. Lentiviral particles were produced by co-transfecting 293T packaging cells with the transfer vector, packaging plasmid, and envelope plasmid using Lipofectamine 3000 (Invitrogen, USA), following the manufacturer's protocol. After 48 hours, viral supernatants were collected and filtered through a 0.45 μm syringe filter to remove cellular debris. Target cells were infected with the viral supernatants in the presence of 8 μg/mL polybrene (Sigma-Aldrich) to enhance transduction efficiency. To select stably transduced cells, G418 (Sigma-Aldrich) or puromycin (Thermo Fisher Scientific, Waltham, MA, USA) was added to the culture medium at appropriate concentrations. After 2 weeks of selection, individual resistant clones were isolated and expanded. The expression levels of ITGB3 and L1CAM in the generated cell lines and their corresponding exosomes were validated by immunoblot analysis.

Immunoblot analysis

Lysate cells and exosomes were prepared in RIPA lysis buffer (Millipore, 20-188; 0.5 M Tris-HCl, pH 7.4, 1.5 M NaCl, 2.5% deoxycholic acid, 10% NP-40, 10 mM EDTA) to extract proteins. The protein concentration was quantified at approximately 20 mg using the Bradford

method. The extracted proteins were subjected to SDS-PAGE and subsequently transferred to PVDF membranes (Invitrogen) for analysis. The membranes were probed with the following primary antibodies: Integrin β1 (1:1000, Cell Signaling Technology Cat# 9699, RRID:AB_11178800), Integrin β3 (1:1000, Cell Signaling Technology Cat# 13166, RRID:AB_2798136), Integrin β4 (1:1000, Cell Signaling Technology Cat# 14803, RRID:AB_2798620), Integrin alpha V (1:1000, Abcam Cat# ab179475, RRID:AB_2716738), NCAM-L1 (1:1000, Santa Cruz Biotechnology Cat# sc-53386, RRID:AB_628937), E-cadherin (1: 1000, Santa Cruz Biotechnology Cat# sc-71008, RRID:AB_1121199), Vimentin (1:1000, Cell Signaling Technology Cat# 5741, RRID:AB_10695459), β-actin (1:3000, Santa Cruz Biotechnology Cat# sc-47778, RRID:AB_626632), TSG101 (1:1000, Abcam Cat# ab125011, RRID:AB_10974262), HSP70 (1:3000, BD Biosciences Cat# 610607, RRID:AB_397941), and HSP90 (1:3000, BD Biosciences Cat# 610418, RRID:AB_397798). The membranes were then treated with a horseradish peroxidase-conjugated secondary antibody. All signals from the membranes were detected using a chemiluminescence system kit (PerkinElmer, Waltham, MA, USA).

In vivo **evaluation of BBB permeability**

To assess the impact of exosomes on BBB permeability, an Evans Blue dye extravasation assay was performed using 6-week-old female C57BL/6 mice (RRID:MGI:2159769). The mice were injected intracardially with exosomes (2×10^9 particles in 100 μL PBS) every other day for a total of three administrations. Twenty-four hours after the final injection, the mice received 2% Evans Blue dye (4 mL/kg) via tail vein injection. Following an 18-hour circulation period, the mice were anesthetized and perfused transcardially with cold PBS via the left ventricle until the perfusate ran clear, ensuring the removal of intravascular dye. Whole brains were carefully harvested and imaged using an IVIS Spectrum (PerkinElmer, RRID:SCR_025239) with

excitation/emission filters set to 620/680 nm. Fluorescence intensity within the brain region was quantified using Living Image software (PerkinElmer) and reported as radiant efficiency. For histological validation of BBB disruption, brain tissues were post-fixed in 4% paraformaldehyde, paraffin-embedded, and sectioned at a thickness of 4 μm. Evans Blue was not injected due to potential fluorescence interference. Sections were subjected to immunofluorescence staining using antibodies against CD31 (1:100, R&D Systems Cat# AF3628, RRID:AB_2161028) and Occludin (1:200, Thermo Fisher Scientific Cat# 40-4700, RRID:AB_2533468). Images were acquired using a Zeiss LSM 880 confocal microscope (RRID:SCR_020925), and the integrity of endothelial tight junctions was evaluated by visualizing junctional continuity and inter-endothelial gap formation.

Cell culture and *in vitro* vascular permeability assay

The immortalized human cerebral microvascular endothelial cell line (HCMEC/D3; Millipore Cat# SCC066, RRID:CVCL_U985) was cultured as a representative model of the human blood-brain barrier (BBB). The cells were maintained in the EndoGRO™-MV Complete Media Kit (SCME004, Millipore), supplemented with 5% fetal bovine serum and 1 ng/mL fibroblast growth factor-2 (FGF-2; GF003, Millipore). Cell culture flasks were pre-coated with type I rat tail collagen (Cat# 08-115, Millipore), diluted 1:20 in PBS, and incubated at 37 °C for 1 hour. The cells were cultured at 37 °C in a humidified incubator with 5% CO_2 and passaged using Trypsin/EDTA and Neutralizer solutions (R001100 and R002100, Thermo Fisher Scientific, USA) when they reached 80%–90% confluence. Human umbilical vein endothelial cells (HUVECs; PCS-100-010, ATCC, RRID:CVCL_2959) were cultured in Medium 200 (M200-500, Gibco), supplemented with Large Vessel Endothelial Supplement (LVES; A1460801, Thermo Fisher), 100 U/mL penicillin, and 100 μg/mL streptomycin. All HUVEC experiments were conducted between passages 2 and 5. Mycoplasma contamination

was assessed using the MycoProbe Mycoplasma Detection Kit (R&D Systems, USA) prior to cryopreservation.

For *in vitro* vascular permeability assays, both hCMEC/D3 and HUVECs were seeded at a density of 5×10^4 cells per insert into collagen-coated Transwell® permeable inserts provided in the Millipore In Vitro Vascular Permeability Assay Kit (ECM642, Millipore). After a 72-hour incubation, confluent endothelial monolayers were confirmed microscopically. Following repeated treatments with test exosomes or control media, an FITC-dextran solution (product number 90328, Millipore) diluted in basal media was added to the upper chamber (75 μL per insert), while 250 μL of basal media was added to the lower chamber. After 20 min of incubation at room temperature, the inserts were removed, and the fluorescence intensity of the lower chamber medium was quantified using a fluorescence microplate reader (excitation: 485 nm, emission: 535 nm) to assess endothelial permeability.

Exosome fluorescent labeling and endothelial cell uptake analysis

Purified exosomes derived from H1975, H1975-L1CAM, and H1975/OR cells were fluorescently labeled using the ExoGlow™ Membrane EV Labeling Kit (System Biosciences; cat. no. EXOGM600A-1) according to the manufacturer's instructions. Briefly, isolated exosomes were incubated with the membrane labeling dye (excitation/emission: 465/635 nm) for 15–30 min at room temperature in the dark. Unbound dye was removed using ExoQuick-based purification, followed by washing steps to ensure minimal background signal. HCMEC/D3) and HUVECs were seeded in 6-well plates and cultured to 70%–90% confluence prior to treatment. Cells were incubated with labeled exosomes at a concentration corresponding to approximately 1 × 10^5 particles per cell for 2 hours. After incubation, cells were washed three times with PBS to remove unbound exosomes and maintained in phenol red–free medium. Nuclei were counterstained with Hoechst 33342 prior to imaging.

Fluorescence images were acquired using a confocal fluorescence microscope, and exosome uptake was quantified based on fluorescence intensity.

Recombinant protein masking of exosomal L1CAM and immunoprecipitation

To mask potential binding sites of exosomal L1CAM, recombinant human NCAM1/CD56 Fc (R&D Systems; cat. no. 10136-NC) or recombinant human Contactin-2/TAG1 (CNTN2; R&D Systems; cat. no. 1714-CN) proteins were incubated with purified exosomes. Briefly, 2×10^9 exosome particles were incubated with the indicated recombinant proteins under gentle rotation to allow binding. For immunoprecipitation assays, exosome–protein complexes were incubated with protein A/G agarose beads (for NCAM1-Fc) or Ni-NTA agarose beads (for His-tagged CNTN2), followed by washing steps to remove unbound material. Bound proteins were eluted and subjected to western blot analysis to confirm the selective binding of recombinant NCAM1 or CNTN2 to exosomal L1CAM. Masked exosomes were subsequently used in functional assays, including *in vitro* BBB permeability experiments, to assess the impact of NCAM1 or CNTN2 masking on exosomal L1CAM-mediated endothelial responses.

Patients and study approval

A total of 81 patients diagnosed with lung cancer at Asan Medical Center (Seoul, Republic of Korea) were enrolled in this study. Patients were classified into three groups based on clinical staging and the presence of brain metastasis (BrM): stage I lung cancer without metastasis (n=27), stage IV lung cancer without BrM (Non-BrM, n=27), and stage IV lung cancer with confirmed BrM (BrM, n=27). All diagnoses were confirmed by histopathological evaluation according to the World Health Organization classification of lung tumors. The study protocol was approved by the Institutional Review Board of Asan Medical Center (approval number: 2016-0692), and written informed consent was obtained from all patients prior to study

participation.

Enzyme-linked immunosorbent assay

Exosomal L1CAM and ITGB3 protein levels were measured using the human L1CAM ELISA Kit (EH290RB, Thermo Fisher Scientific) and the human ITGB3 ELISA Kit (MBS2516199, MyBioSource), following the manufacturers' protocols. Exosome samples were diluted in assay buffer, loaded into antibody-coated wells, and incubated sequentially with detection antibodies, streptavidin-HRP, and TMB substrate. Absorbance was measured at 450 nm using a microplate reader. Protein concentrations were calculated using standard curves and normalized to the number of exosome particles. All samples were analyzed in duplicate and repeated in independent experiments.

Diagnostic Modeling

To evaluate the diagnostic performance of exosomal biomarkers, receiver operating characteristic (ROC) curve analysis was performed using patient-derived serum samples. The area under the ROC curve (AUC) was calculated to assess the discriminative power of each marker and the combination panels. ROC curves were generated using the pROC package in R (version 4.2.2, RRID:SCR_024286), and the statistical significance of AUC values was evaluated based on the method proposed by Mason and Graham, with p-values < 0.05 considered significant. For each biomarker, exosomal protein expression levels were normalized to particle number and log-transformed prior to analysis. Sensitivity and specificity were computed across a range of cutoff values, and Youden's index was used to determine the optimal threshold. Comparisons between models (e.g., single markers vs. combined panels) were conducted using DeLong's test for correlated ROC curves.

Machine learning-based validation of biomarker performance

To further strengthen the comparison between individual biomarkers and the combined panel

under limited sample size conditions, machine learning-based validation analyses were performed using exosomal ITGB3 and L1CAM protein levels quantified by ELISA. Three feature configurations were evaluated: ITGB3 alone, L1CAM alone, and the combined ITGB3+L1CAM panel. Two independent nonlinear machine learning algorithms—Random Forest and Extreme Gradient Boosting (XGBoost)—were applied due to their distinct modeling structures and optimization principles. To minimize overfitting and assess reproducibility, repeated internal validation strategies were implemented. Specifically, 5-fold cross-validation was repeated 30 times with random resampling of the dataset. In addition, bootstrap resampling was performed for 300 iterations to further estimate model stability and expected performance variability. For each validation run, ROC curves were generated, and the AUC was calculated to quantify discriminative performance. Mean AUC values across all repetitions were used for model comparison. The consistency of model ranking among the combined panel and single-marker models was assessed across validation iterations and algorithms. All analyses were conducted using identical validation frameworks for both Random Forest and XGBoost models to ensure comparability. The results of these machine learning-based validation analyses are presented in Supplementary Fig. S13.

Supplementary Figure S1

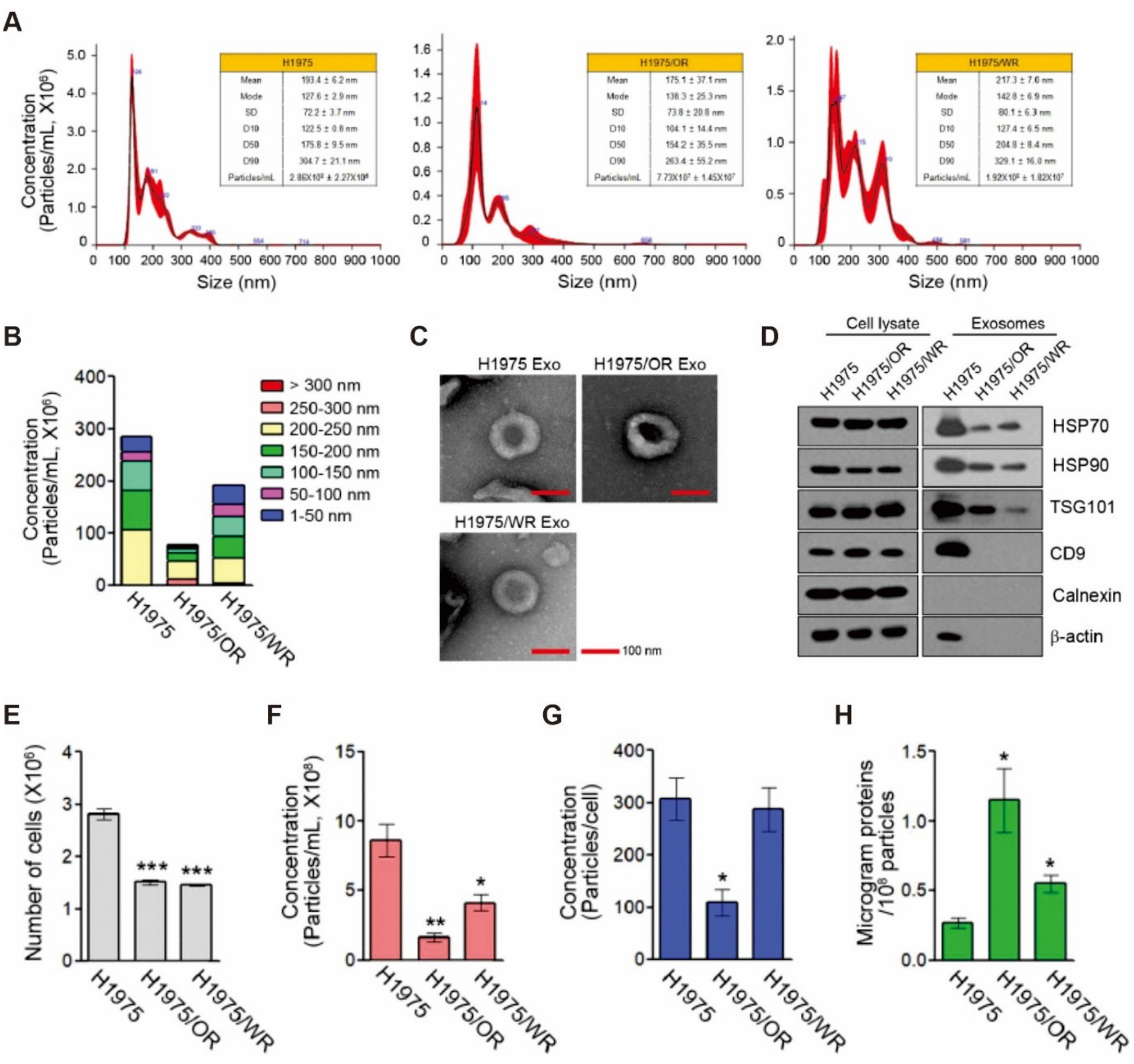

Fig. S1. Characterization of exosomes derived from NSCLC cell lines. (**A**) Size distribution and concentration of the isolated particles were determined using nanoparticle tracking analysis. (**B**) Stacked bar graph showing particle counts according to exosome size. (**C**) Representative transmission electron microscopy images displaying the morphology of exosomes derived from each cell line. (**D**) Immunoblot analysis of cell lysates and corresponding cell-derived exosomes was conducted to assess the expression of exosomal markers. (**E**) Bar graph indicating the number of cells at the time of exosome collection. (**F**) Total particle count of exosomes isolated from each condition. (**G**) Exosome particle count was

normalized to the number of cells. (**H**) Protein concentration per exosome particle. Statistical significance: *$p < 0.05$; **$p < 0.005$; ***$p < 0.0005$.

Supplementary Figure S2

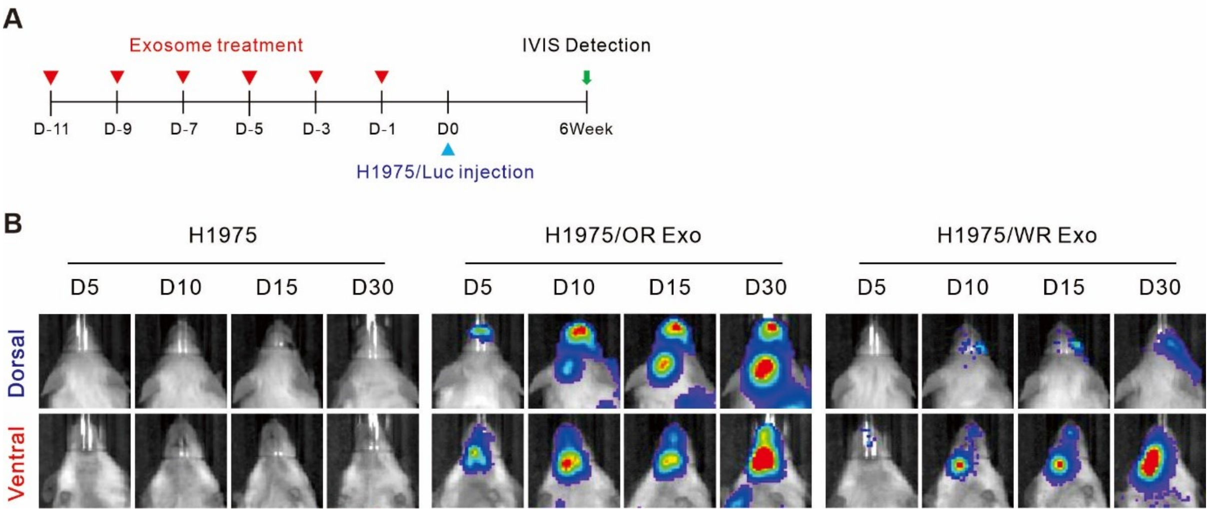

Fig. S2. Exosome education model for confirming metastasis patterns to the brain. (**A**) Schematic diagram of the lung cancer cell-derived exosome education model for metastasis detection. (**B**) Representative IVIS images at each time point of the mouse brain injected with H1975/Luc cells following each exosome training.

Supplementary Figure S3

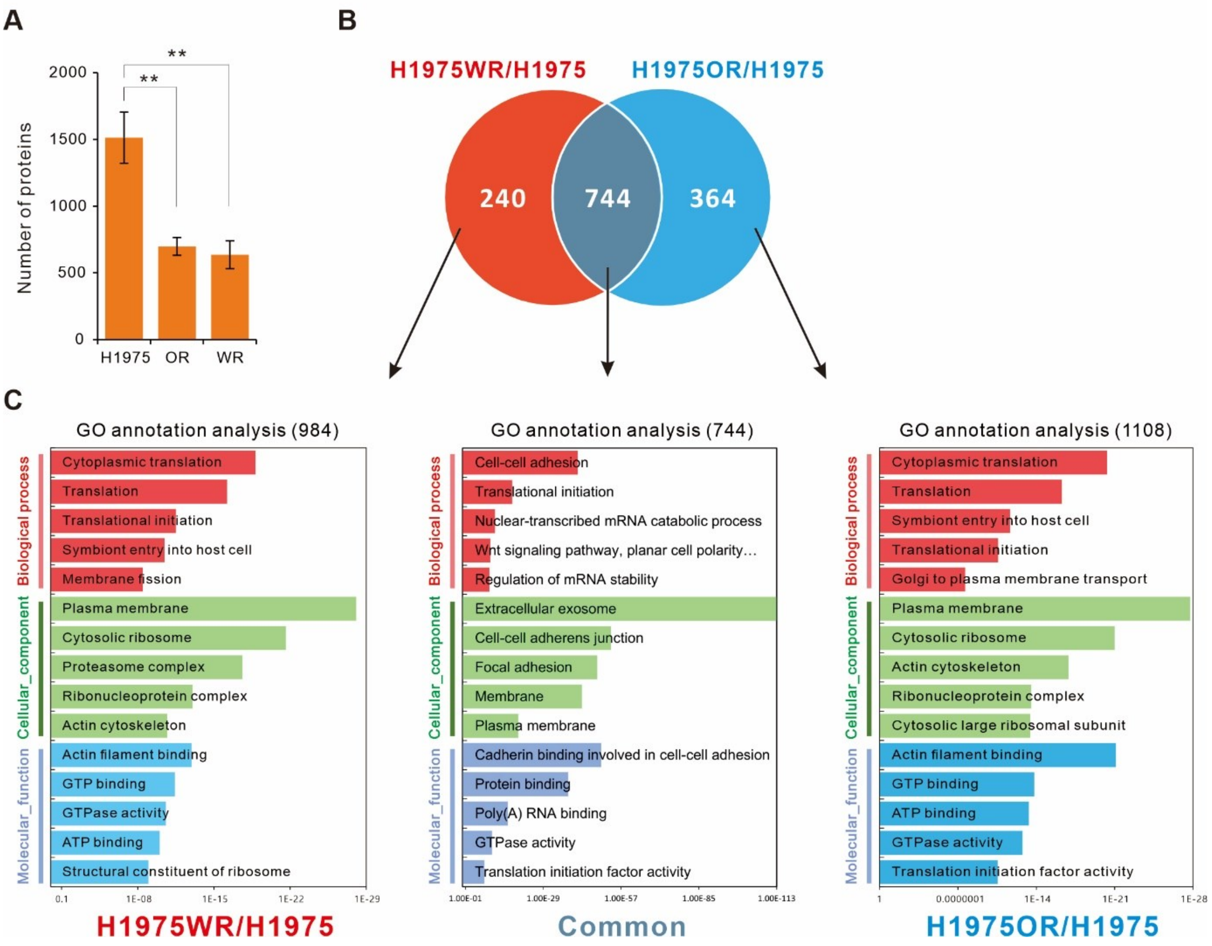

Fig. S3. Stepwise proteomic analysis for identification of common differentially expressed exosomal proteins. LC–MS/MS-based proteomic analysis was performed using exosomes isolated from parental H1975 cells and two resistant sublines, H1975/OR and H1975/WR. (A) Bar graph showing the total number of exosomal proteins identified in H1975, H1975/OR, and H1975/WR cells. (B) Venn diagram illustrating the number of significantly regulated exosomal proteins identified by pairwise comparisons of H1975/OR vs H1975 and H1975/WR vs H1975. The overlap represents commonly altered proteins shared between the two resistant sublines. (C) Gene Ontology (GO) enrichment analysis based on differentially expressed exosomal proteins identified in H1975/WR vs H1975 (984 proteins), H1975/OR vs H1975 (1108 proteins), and their common intersection (744 proteins).

Supplementary Figure S4

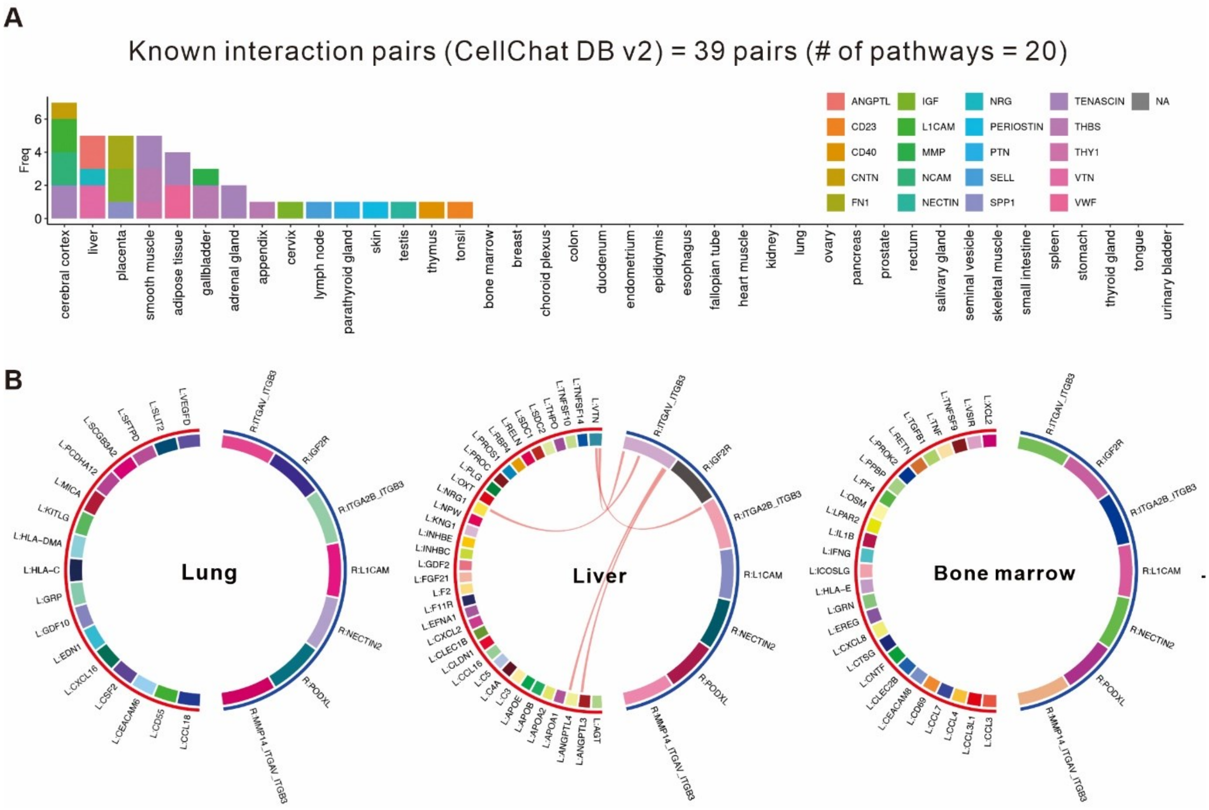

Fig. S4. Predicted interaction network between exosomal membrane proteins and tissue-specific target proteins using the CellChat v2 database. (**A**) Interaction network graph illustrating organ-specific ligand–receptor interactions between 74 upregulated exosomal membrane proteins (identified in H1975/OR and H1975/WR exosomes relative to H1975 exosomes) and 621 predicted target proteins across various organs. (**B**) Circos plot depicting predicted interactions between seven representative exosomal membrane proteins and target proteins reported to be expressed in the lung, liver, and bone marrow. Red lines in the center indicate specific protein–protein interactions.

Supplementary Figure S5

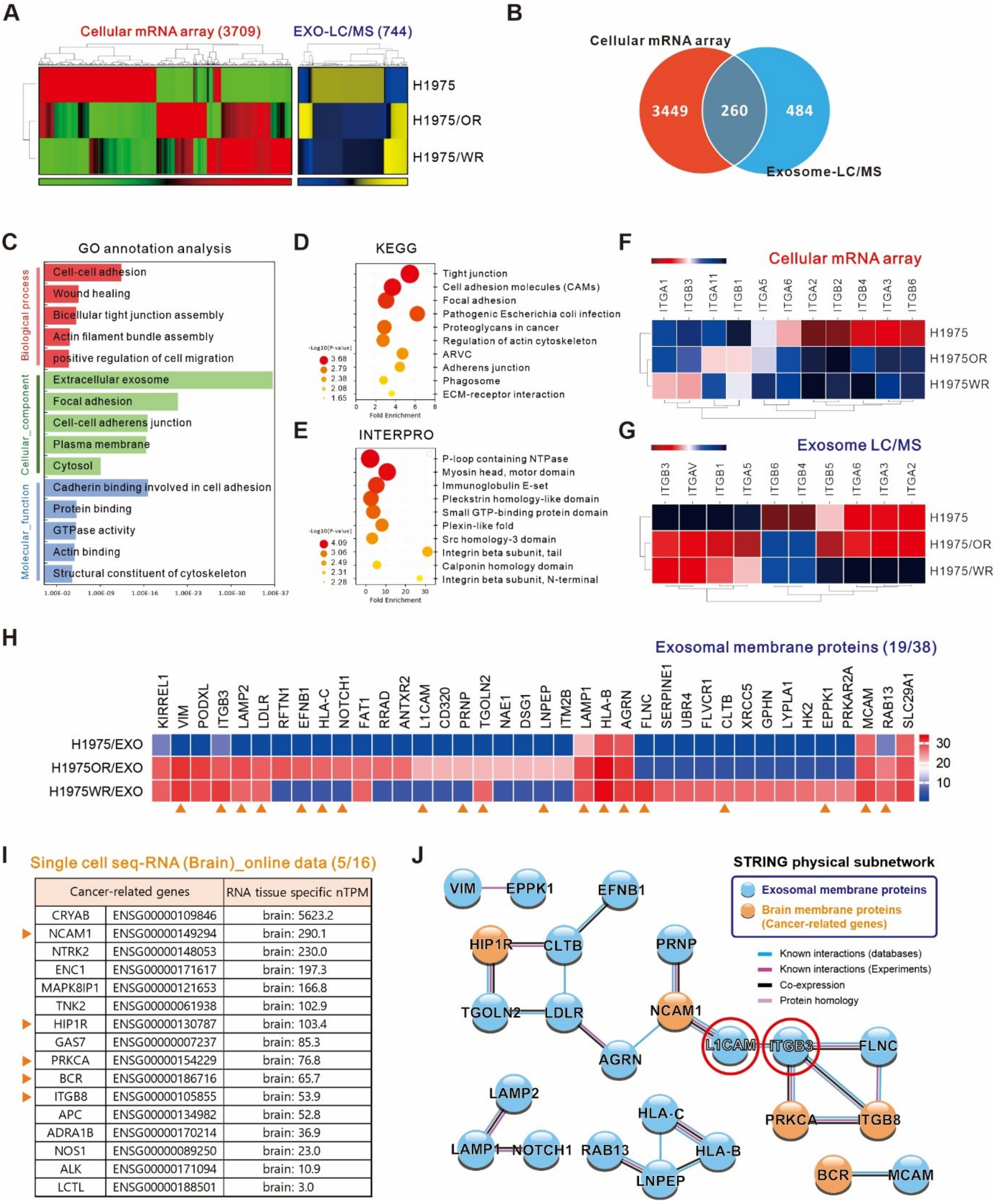

Fig. S5. Integrated transcriptomic and proteomic analysis in metastatic lung cancer cell lines. (**A**) Heatmap showing differentially expressed genes from cellular mRNA sequencing and differentially expressed proteins from exosome LC-MS/MS analysis across the parental H1975, osimertinib-resistant H1975/OR, and WZ4002-resistant H1975/WR cell lines. (**B**) Venn diagram depicting the overlap of significantly upregulated and downregulated genes/proteins among the groups. A total of 260 overlapping molecules were identified ($P < 0.05$; fold change > 2). (**C–E**) Functional enrichment analyses of the overlapping molecules using GO biological processes (C), KEGG pathways (D), and INTERPRO protein domain classification (E). (**F, G**) Expression levels of integrin family members shown in heatmaps based on cellular mRNA expression (F) and exosomal proteomic data (G). (**H**) Heatmap of 38 exosomal membrane proteins that demonstrated significant upregulation in exosomes derived from resistant cells (H1975/OR and H1975/WR) compared to parental H1975 cells. Orange arrows indicate proteins known to interact with brain-specific membrane proteins. (**I**) Single-cell RNA sequencing (scRNA-seq) data extracted from the Human Protein Atlas (HPA) identifying 16 cancer-associated genes that are highly expressed in brain tissue and localized to the plasma membrane. Orange arrows highlight genes predicted to interact with exosomal membrane proteins. (**J**) Physical interaction network analysis showing predicted associations between upregulated exosomal membrane proteins (blue circles) and brain-specific membrane proteins (orange circles). Key exosomal membrane proteins with confirmed interactions are highlighted with red outlines.

Supplementary Figure S6

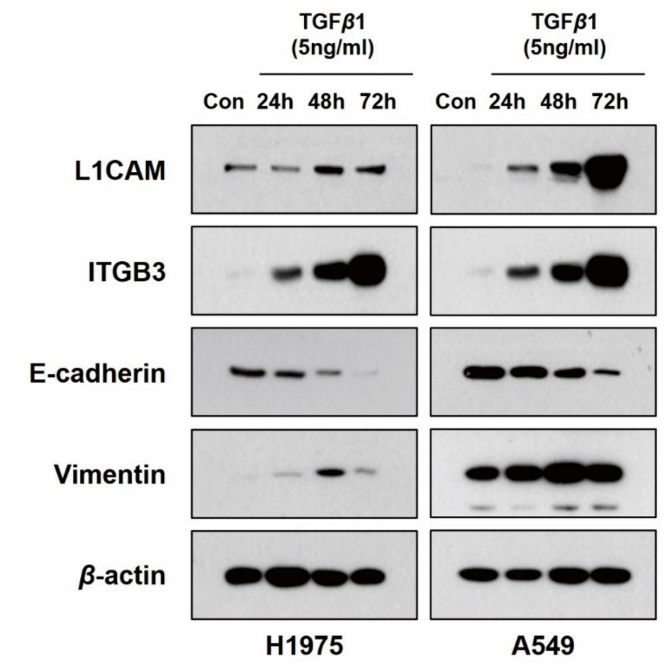

Fig. S6. Expression of L1CAM and ITGB3 following EMT induction in NSCLC cells. Immunoblot analysis of L1CAM and ITGB3 expression in H1975 (EGFR-mutant) and A549 (EGFR wild-type) non-small cell lung cancer (NSCLC) cell lines treated with TGF-β1 (5 ng/mL) for 24, 48, and 72 hours. This experiment was conducted to assess the time-dependent changes in expression of these proteins during the induction of epithelial-to-mesenchymal transition.

Supplementary Figure S7

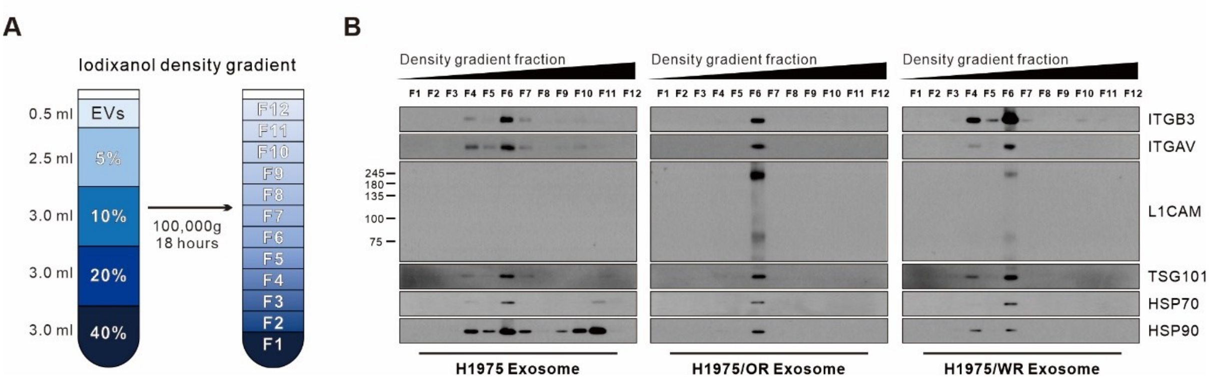

Fig. S7. Detection of L1CAM and integrin expression in exosomes isolated via iodixanol density gradient ultracentrifugation. (**A**) Schematic representation of the iodixanol density gradient ultracentrifugation protocol used to fractionate exosomes derived from H1975, H1975/OR, and H1975/WR cells. Exosome suspensions were layered onto a discontinuous iodixanol gradient (5%, 10%, 20%, 40%) and ultracentrifuged at 100,000 × g for 18 hours at 4 °C. (**B**) Sequential fractions (F1–F12) collected from the top of the gradient were analyzed by immunoblotting to assess the expression levels of L1CAM, integrins (ITGB3 and ITGAV), and exosome markers (TSG101, HSP70, and HSP90), confirming the enrichment of target proteins in exosome-rich fractions.

Supplementary Figure S8

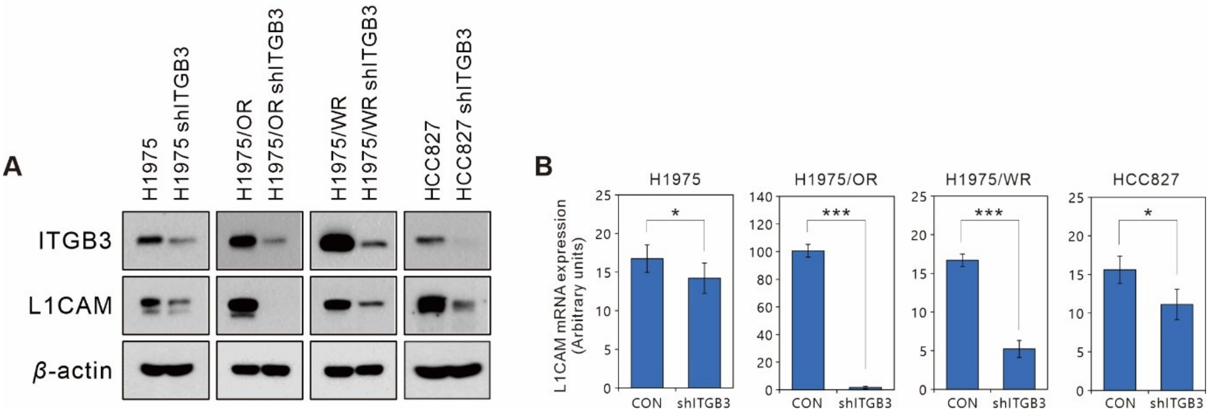

Fig. S8. Downregulation of L1CAM expression following ITGB3 knockdown in NSCLC cell lines. (**A**) Immunoblot analysis of ITGB3 and L1CAM expression was conducted in H1975, H1975/OR, H1975/WR, and HCC827 cells, as well as in their respective ITGB3 knockdown (shITGB3) counterparts. β-actin was used as a loading control. (**B**) Quantitative RT-PCR analysis of L1CAM mRNA levels in the same cell lines, showing a significant reduction in transcript levels following ITGB3 silencing. Data represent mean ± SEM; $*p < 0.05$; $***p < 0.0005$.

Supplementary Figure S9

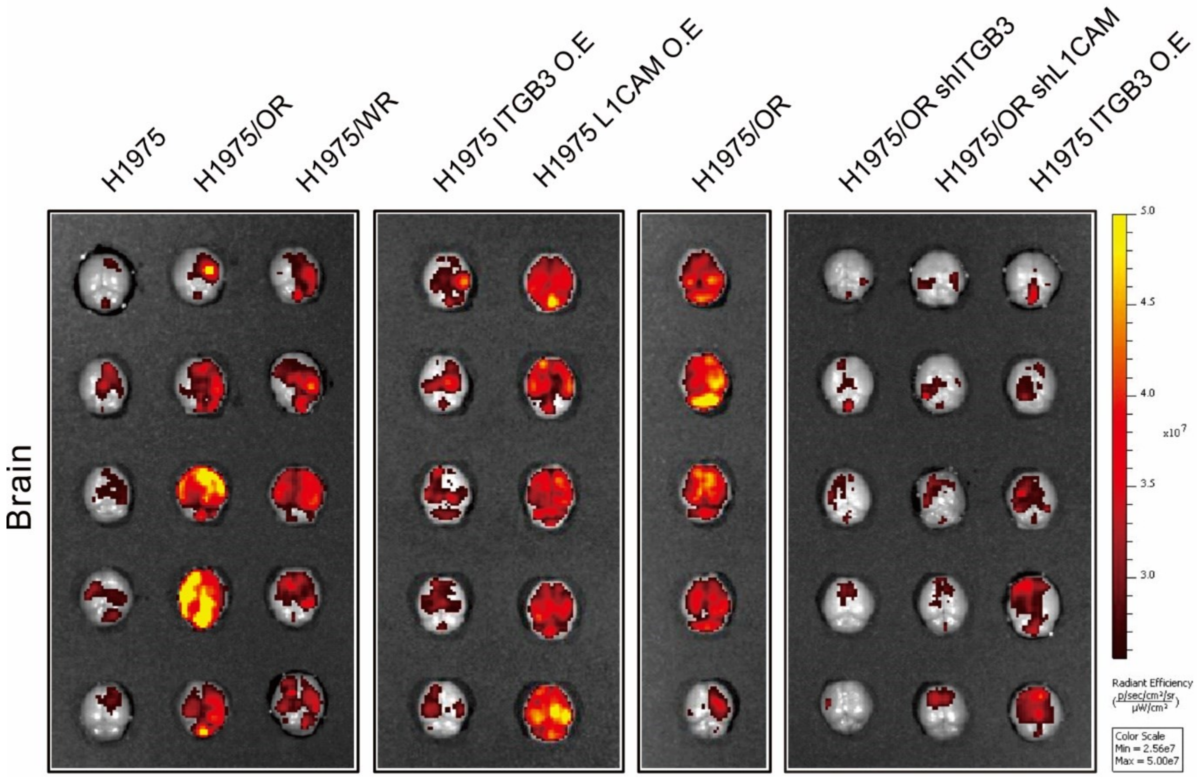

Fig. S9. ***In vivo*** **brain uptake of exosomes modulated by ITGB3 and L1CAM expression.** Near-infrared-labeled exosomes were prepared from various NSCLC cell lines, including H1975, H1975/OR, H1975/WR, H1975-ITGB3 overexpression (O.E), H1975-L1CAM O.E, and H1975/OR cells with ITGB3 or L1CAM knockdown (shITGB3, shL1CAM). Labeled exosomes (2×10^9 particles/100 μL) were injected intracardially into mice. After 24 hours, the mouse brains were harvested, and fluorescence signals were visualized using IVIS imaging. Representative images from five mice per group are shown.

Supplementary Figure S10

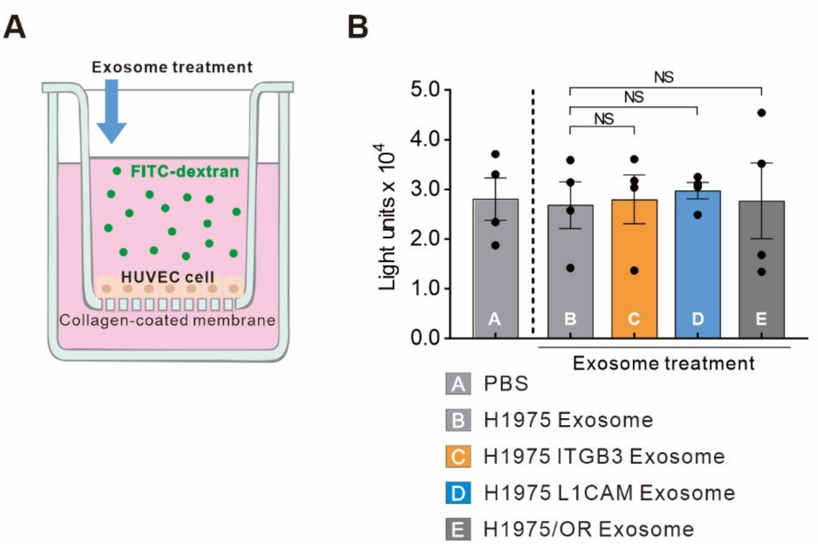

Fig. S10. *In vitro* vascular permeability assay using HUVECs treated with exosomes derived from NSCLC cell lines. (**A**) Schematic diagram of the transwell-based permeability assay. Human umbilical vein endothelial cells were seeded onto collagen-coated membranes within Transwell® inserts and treated with exosomes derived from the indicated NSCLC cell lines. FITC-dextran was added to the upper chamber to evaluate permeability. (**B**) Quantification of FITC-dextran fluorescence in the lower chamber following exosome treatment. Exosomes were derived from H1975, H1975-ITGB3, H1975-L1CAM, and H1975/OR cells. Fluorescence was measured at excitation/emission wavelengths of 485/535 nm. Experiments were performed in quadruplicate (n = 4 chambers per group).

Supplementary Figure S11

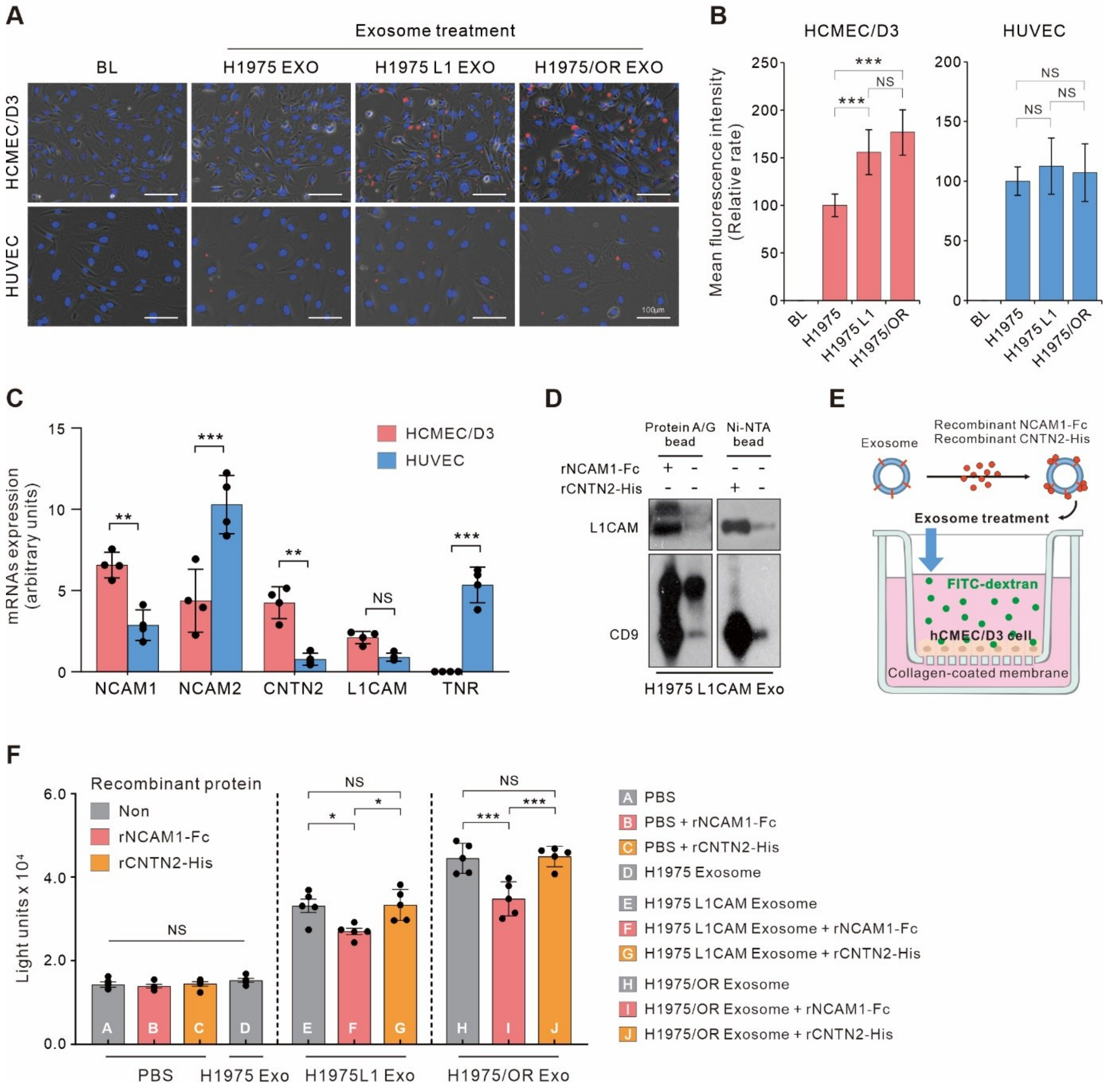

Fig. S11. Brain endothelial-specific uptake and NCAM1-dependent functional interaction of exosomal L1CAM. (**A**) Representative fluorescence images showing uptake of fluorescently labeled exosomes derived from H1975, H1975-L1CAM, and H1975/OR cells by Human BBB cell lines (HCMEC/D3) and human umbilical vein endothelial cells (HUVECs). Exosomes were labeled with a membrane fluorescent dye and incubated with endothelial cells before imaging. (**B**) Representative fluorescence images and quantitative analysis of labeled exosome uptake in HCMEC/D3 and HUVEC cells following treatment with each exosome

group. Fluorescence intensity was quantified per cell. (**C**) Relative mRNA expression levels of five predicted endothelial binding partners of exosomal L1CAM in HCMEC/D3 and HUVEC cells, as determined by quantitative RT-PCR. (**D**) Immunoprecipitation analysis confirming selective binding of recombinant human NCAM1 or CNTN2 proteins to exosomal L1CAM following masking treatment. (**E**) Schematic representation of the *in vitro* BBB permeability assay using HCMEC/D3 cells cultured as monolayers on collagen-coated Transwell inserts. (**F**) Quantification of FITC-dextran permeability across HCMEC/D3 monolayers after treatment with indicated exosome groups, with or without pretreatment using recombinant human NCAM1 or CNTN2 proteins. Data are presented as mean ± standard deviation. *P < 0.05, **P < 0.005, ***P < 0.0005. NS = not significant.

Supplementary Figure S12

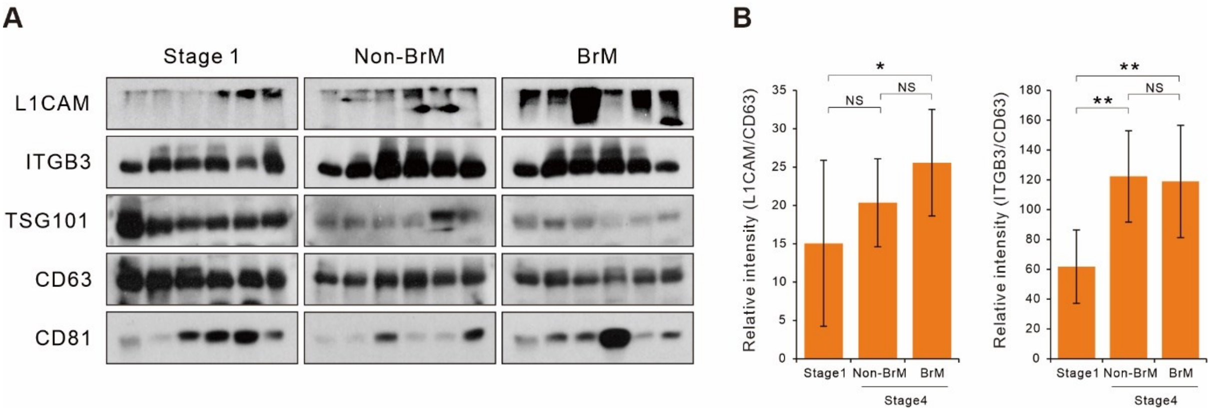

Fig. S12. Validation of patient-derived exosomal ITGB3 and L1CAM expression using western blot analysis. Western blot analysis was performed using serum-derived exosomes from randomly selected patients with lung cancer to validate ELISA-based quantification of exosomal ITGB3 and L1CAM. (**A**) Representative western blot images showing exosomal L1CAM and ITGB3 expression in patient-derived exosome samples. (**B**) Densitometric quantification of L1CAM and ITGB3 protein levels normalized to CD63. Data are presented as mean ± standard deviation. *P < 0.05, **P < 0.005, ***P < 0.0005. NS = not significant.

Supplementary Figure S13

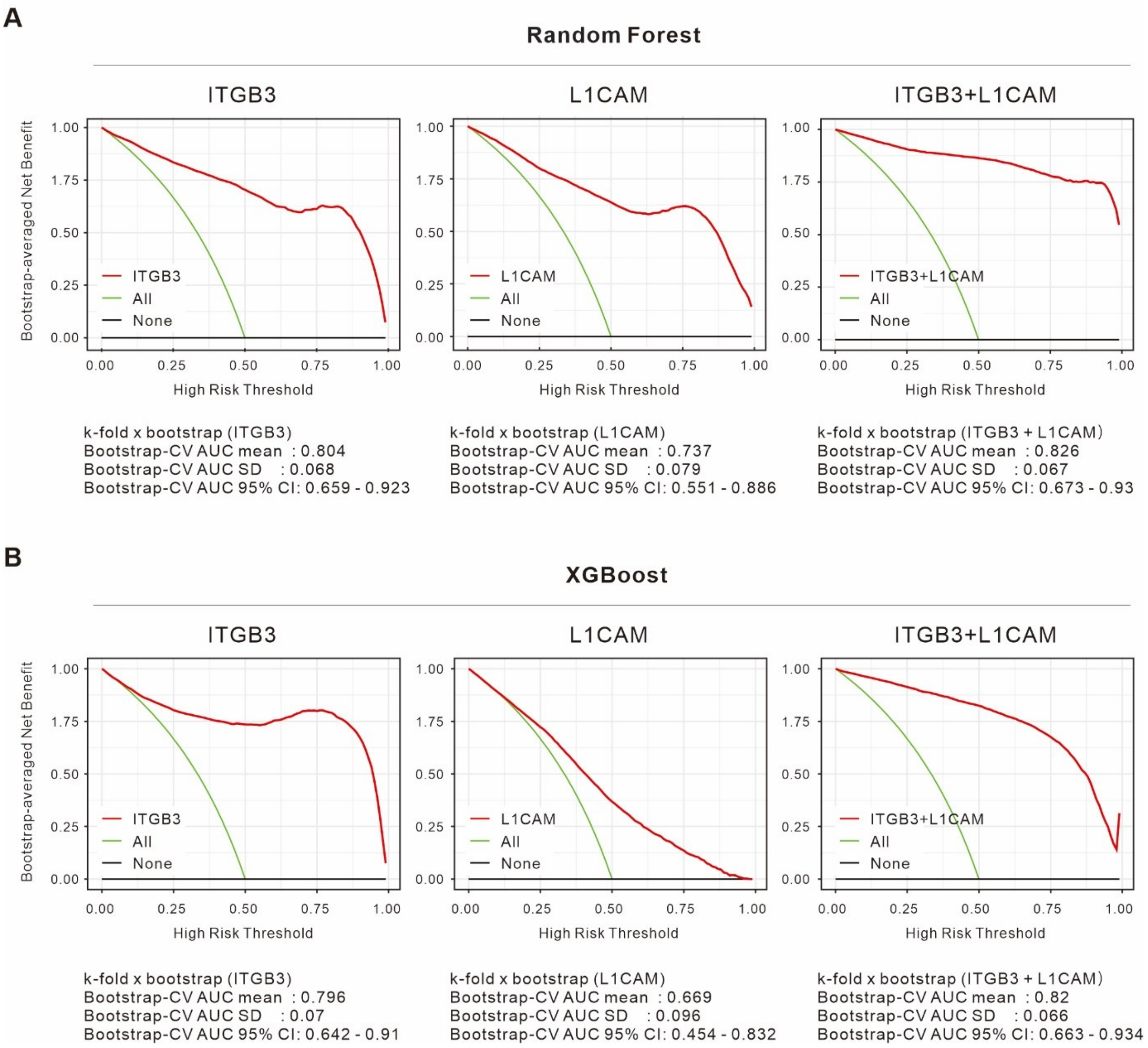

Fig. S13. Machine learning-based validation of ITGB3 and L1CAM biomarker performance. Machine learning-based analyses were performed to evaluate the robustness and reproducibility of biomarker performance under repeated internal validation. (**A**) Random Forest-based classification results comparing ITGB3 alone, L1CAM alone, and the combined ITGB3+L1CAM panel using repeated 5-fold cross-validation and bootstrap resampling. (**B**) XGBoost-based classification results using the same validation framework.

Supplementary Figure S14

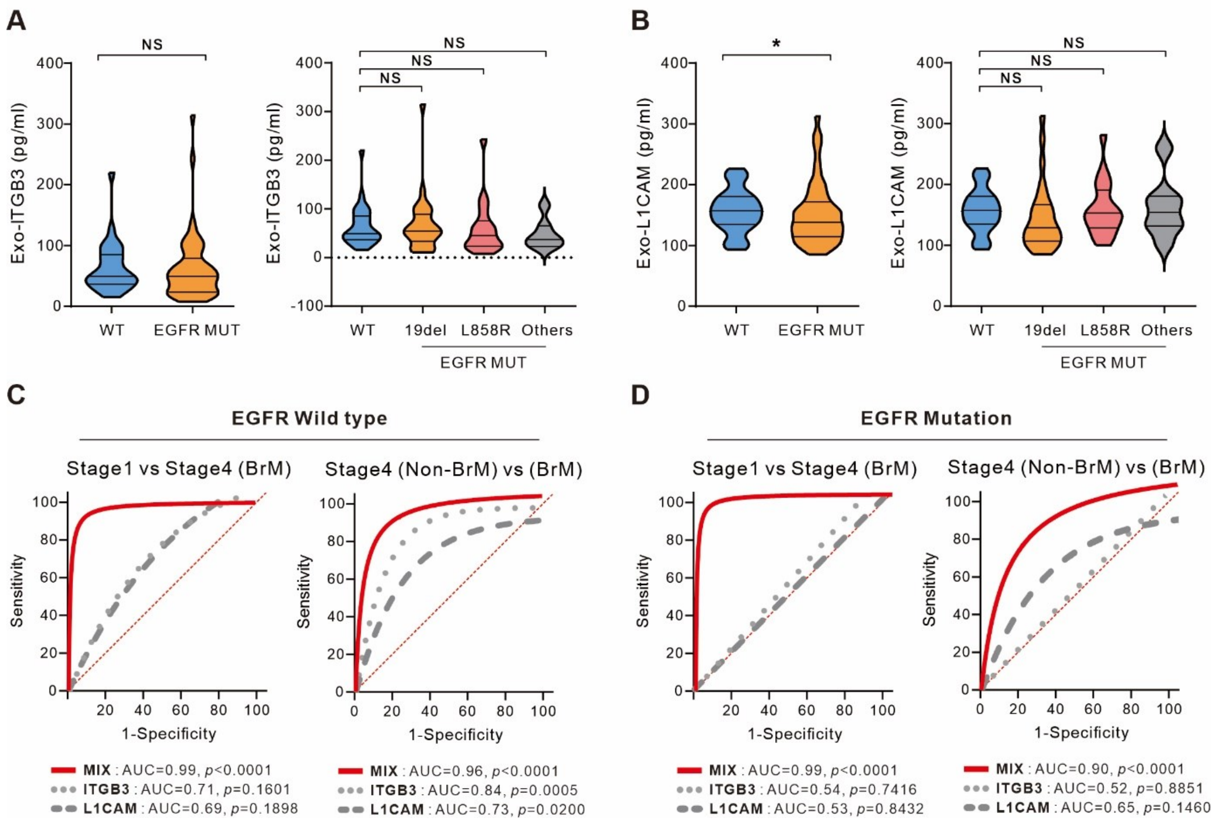

Fig. S14. Stratified analysis of exosomal ITGB3 and L1CAM by EGFR mutation status. (**A**) Comparison of exosomal ITGB3 expression levels between EGFR wild-type and EGFR-mutant patient groups, with further stratification by specific EGFR mutation subtypes. (**B**) Comparison of exosomal L1CAM expression levels between EGFR wild-type and EGFR-mutant patient groups, including subgroup analysis by EGFR mutation subtype. (**C**) Receiver operating characteristic (ROC) analysis of exosomal ITGB3 and L1CAM, individually and in combination, for diagnostic efficacy evaluation in patients with EGFR wild-type lung cancer, including comparisons between Stage I vs Stage IV (BrM) and Stage IV Non-BrM vs Stage IV BrM groups. (D) ROC analysis of exosomal ITGB3 and L1CAM, individually and in combination, for diagnostic efficacy evaluation in patients with EGFR-mutant lung cancer, including comparisons between Stage I vs Stage IV (BrM) and Stage IV Non-BrM vs Stage IV BrM groups. *P < 0.05, NS = not significant.

Supplementary Figure S15

A. Figure 5D

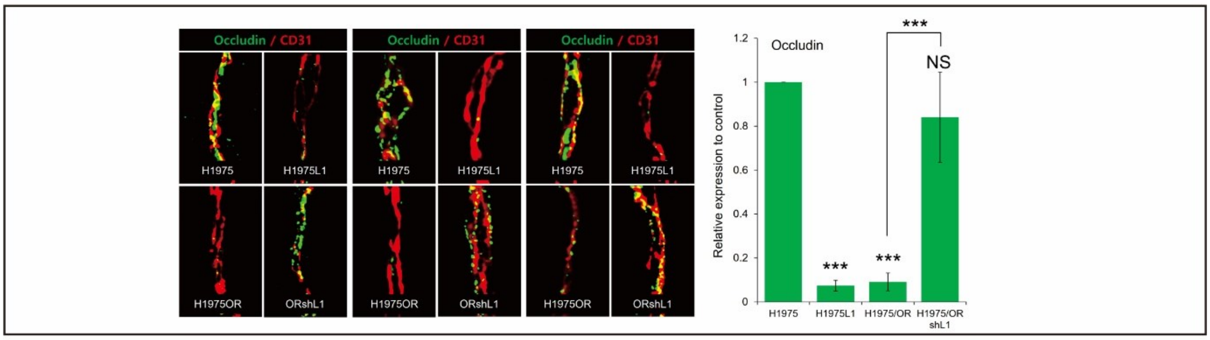

B. Figure 3I

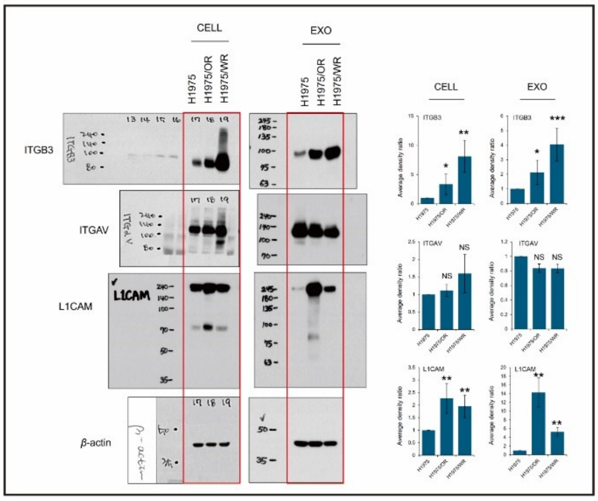

C. Figure 4A and B

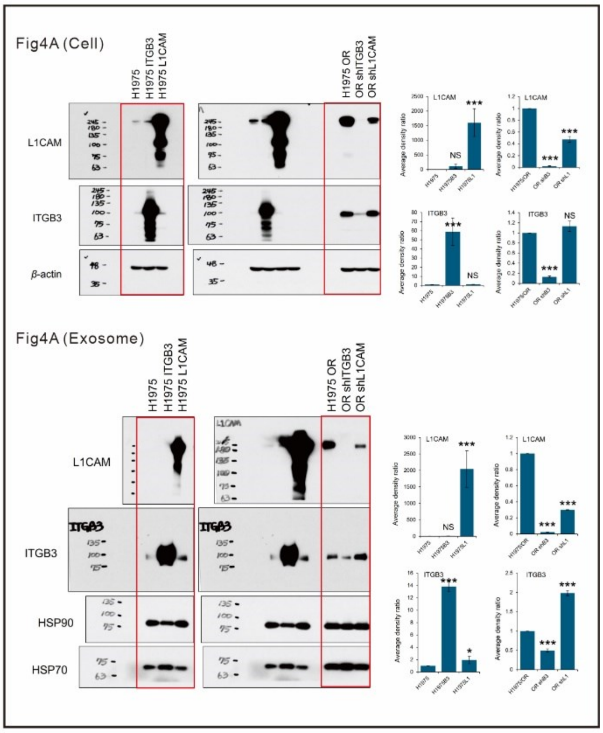

D. Supplementary Figure S1D

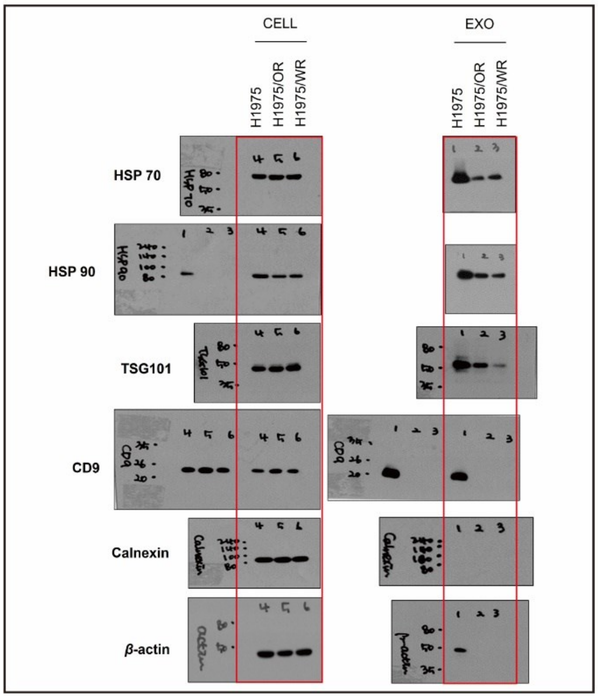

E. Supplementary Figure S6

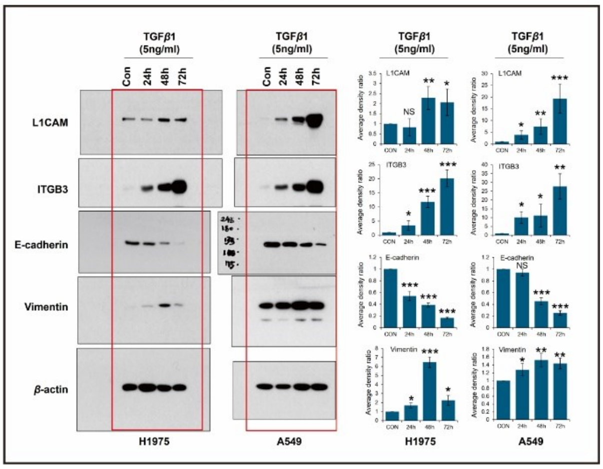

F. Supplementary Figure S7B

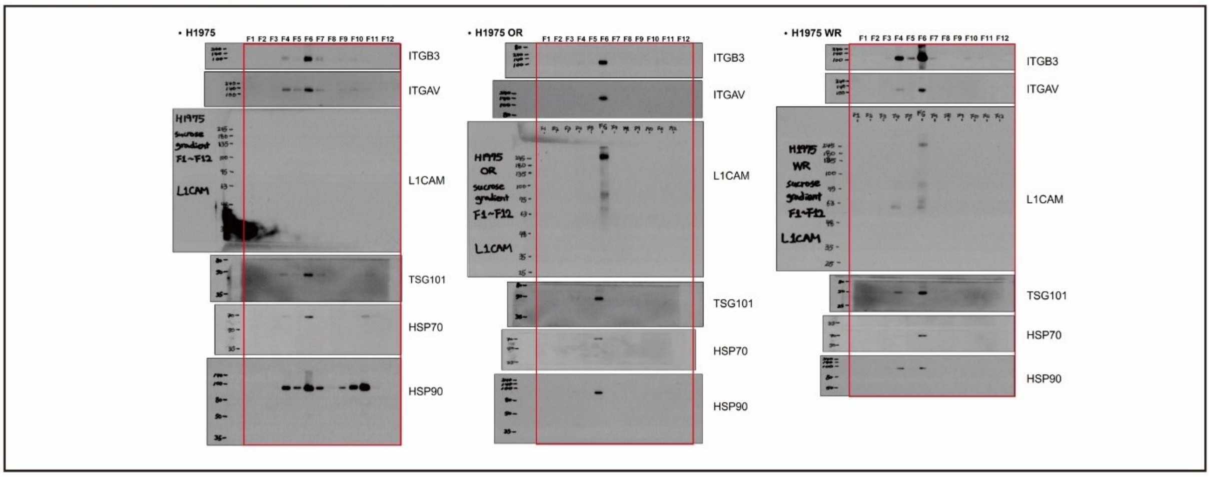

G. Supplementary Figure S8

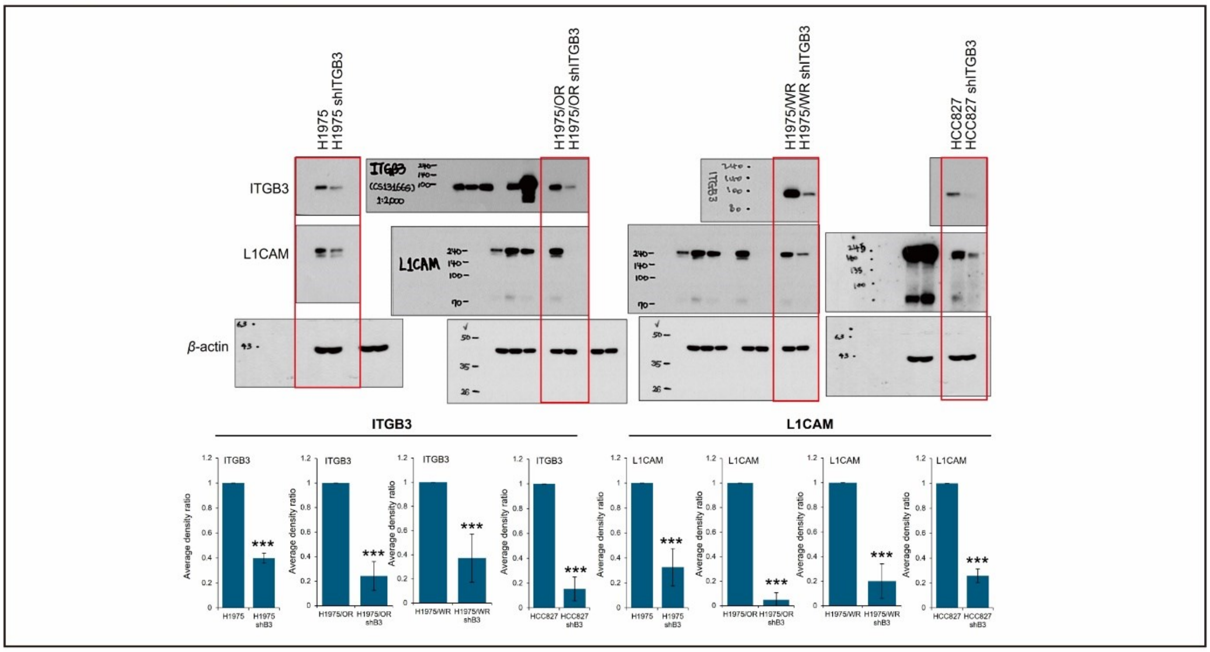

H. Supplementary Figure S11

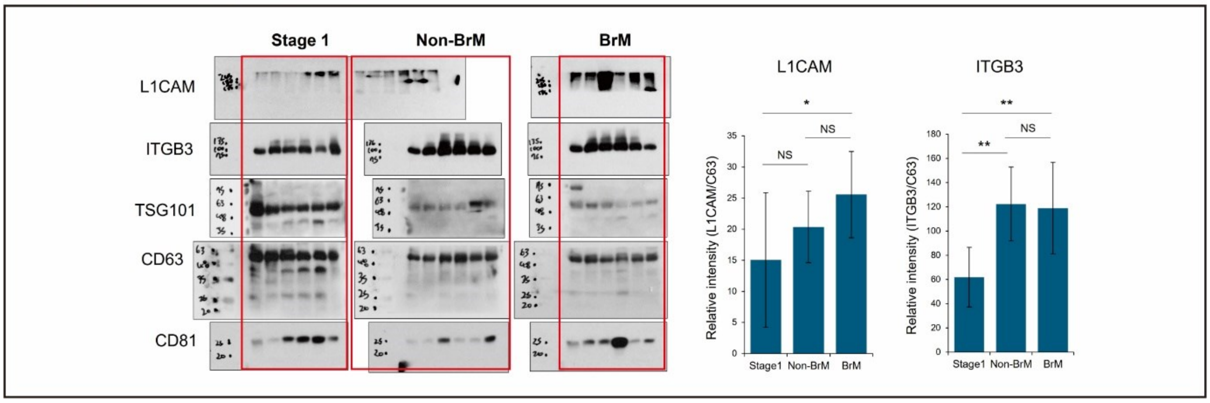

Figure S15. Quantitative analysis and full blot images supporting western blot and immunofluorescence data. Quantitative analyses and full-length western blot (WB) images are provided to support the reproducibility of WB and immunofluorescence (IF) data presented in the main and supplementary figures. Quantification was performed for experiments with at least three independent biological replicates ($n \geq 3$), where applicable. (**A**) Quantification of Occludin immunofluorescence intensity in mouse brain endothelial cells corresponding to Fig. 5D. (**B**) Full-length western blot images and densitometric quantification of proteins shown in Fig. 3I. (**C**) Full-length western blot images and densitometric quantification of proteins shown in Fig. 4A and B. (**D**) Full-length western blot images corresponding to Supplementary Fig. S1D, provided to show representative exosome marker expression without quantitative analysis. (**E**) Full-length western blot images and densitometric quantification of proteins shown in Supplementary Fig. S6. (**F**) Full-length western blot images corresponding to Supplementary Fig. S7B, provided as representative exosome marker blots without quantitative analysis. (**G**) Full-length western blot images and densitometric quantification of proteins shown in Supplementary Fig. S8. (**H**) Full-length western blot images and densitometric quantification of proteins shown in Supplementary Fig. S11. For quantified datasets, densitometric analyses were performed using normalized band intensities relative to the indicated loading controls, and data are presented as mean ± standard deviation from at least three independent experiments.
